# Dynamic ammonia exchange within a mixed deciduous forest canopy in the Southern Appalachians

**DOI:** 10.1016/j.ecolmodel.2024.111007

**Published:** 2025-02-01

**Authors:** Rick D. Saylor, John T. Walker, Zhiyong Wu, Xi Chen, Donna B. Schwede, A.Christopher Oishi, Nebila Lichiheb

**Affiliations:** a National Oceanic and Atmospheric Administration, Air Resources Laboratory, Oak Ridge, TN 37830, United States; b U. S. Environmental Protection Agency, National Risk Management Research Laboratory, Research Triangle Park, NC 27711, United States; c U. S. Environmental Protection Agency, National Exposure Research Laboratory, Research Triangle Park, NC 27711, United States; d U. S. Forest Service, Southern Research Station, Otto, NC 28763, United States; e Oak Ridge Associated Universities, Oak Ridge, TN 37830, United States

**Keywords:** Deposition, Dew, Ammonia, Forest canopy, In-canopy

## Abstract

Ammonia (${NH}_{3}$) concentration and flux measurements made in 2016 in a mixed deciduous forest at the western North Carolina Coweeta Hydrologic Laboratory are analyzed using a multi-layer, one-dimensional column model with detailed canopy physics and bi-directional exchange. Simulations for April 26–30 and July 19–30 are presented to assess the model’s ability to represent measured in-canopy ${NH}_{3}$ profiles and probe the processes that control bi-directional exchange with the canopy and forest floor. During dry canopy conditions, model simulations are found to well reproduce measured in-canopy profiles for both the April and July periods, given appropriate model inputs. Results from the model, and the shape of in-canopy ${NH}_{3}$ profiles, are sensitive to vertical turbulent mixing, the values of the input soil/litter emission potential, and the assumed litter resistance. ${NH}_{3}$ fluxes simulated above the forest canopy are very small (−25 to −5 ng m^−2^ s^−1^ in April and < 1 ng m^−2^ s^−1^ in July) with primarily deposition to the canopy during the April time period, but with mixed deposition/emission during July. The model also suggests that net deposition or emission of ${NH}_{3}$ can be a function of location within the canopy, depending on the difference between the air concentration and the effective canopy compensation point. However, during periods when the canopy is wet from overnight dew and drying rapidly, the model does a poor job of replicating in-canopy profiles, typically underestimating ${NH}_{3}$ concentrations, since the model does not account for the release of ${NH}_{3}$ from evaporating dew. Although available data during the field campaign are not sufficient to rule out other potential hypotheses, given that the model reasonably reproduces in-canopy profiles during dry canopy periods, but fails during periods of rapid drying, the results are suggestive that dew is playing a major role in ${NH}_{3}$ concentration changes observed in July during the field study. Additional studies and measurements are needed to determine the processes and environmental controls that affect ${NH}_{3}$ absorption and release from dew and to evaluate the importance of this process for modeling deposition and re-emission on the regional scale. Further questions that arise from our findings are whether the variation of ${NH}_{3}$ deposition or emission with location in the canopy is important from an ecological perspective and how in-canopy dynamics might be represented in regional-scale air quality models. Traditional big-leaf approaches of modeling ${NH}_{3}$ bi-directional exchange cannot account for in-canopy variation such as that presented here, and so multi-layer approaches may need to be developed for more nuanced estimates of ${NH}_{3}$ deposition to forest ecosystems.

## 1. Introduction

Ammonia (${NH}_{3}$) is an important component of the nitrogen cycle and globally accounts for a large fraction of anthropogenic emissions of reactive nitrogen species (Fowler et al., 2013). Through its gaseous reactions with atmospheric acids, ${NH}_{3}$ influences the atmospheric concentration of fine particles and thereby impacts visibility, weather, climate and human health. Excessive deposition of ${NH}_{3}$, in either its gaseous or particle form, can have harmful effects on terrestrial and aquatic ecosystems (Erisman et al., 2013). Globally, emissions of ${NH}_{3}$ are expected to increase through the end of this century due to increasing fertilizer use necessary to feed a growing world population (Bauer et al., 2016).

The bi-directional nature of the interactions of ${NH}_{3}$ between plant canopies and the atmosphere has been recognized for quite some time (Farquhar et al., 1979, 1980; Lemon and Van Houtte, 1980). Accounting for the bi-directionality of ${NH}_{3}$’s interactions with plants in models presents difficult challenges and has resulted in a variety of approaches when using models to analyse field measurements (e.g., Nemitz et al., 2000, 2001; Walker et al., 2013) or in attempting to represent bi-directionality in air quality models (e.g., Wu et al., 2009; Pleim et al., 2013). Although there have been many ${NH}_{3}$ flux measurement campaigns over agricultural and natural grassland ecosystems, there have been fewer conducted over forested areas. Of the studies that have been performed, nearly all report on the bi-directional nature of measured ${NH}_{3}$ fluxes over forests, regardless of forest type, but with deposition or emissions predominating based primarily on the magnitude of the ambient ${NH}_{3}$ concentration and/or the presence/absence of water in the canopy. Langford and Fehsenfeld (1992) measured ${NH}_{3}$ concentrations with molybdenum oxide annular denuders on the western and eastern edges of Roosevelt National Forest in Colorado. The forest, located between the highly agricultural High Plains to the east and the relatively natural Front Range of the Colorado Mountains to the west, was found to be either a sink or source of ${NH}_{3}$, depending upon the upwind ${NH}_{3}$ concentration. When the wind was from the west and ${NH}_{3}$ concentrations were very low, the forest acted as a source, producing higher concentrations on the downwind side of the forest. Alternatively, when an east wind brought higher ${NH}_{3}$ concentrations from agricultural sources on the plains, the forest acted as a sink, removing ${NH}_{3}$ and producing lower concentrations on the westward side of the forest.

Wyers et al. (1992) measured ${NH}_{3}$ concentrations (vanadium pentoxide annular denuders) and fluxes (gradient method) above a Douglas fir canopy ($h_{c}=14-16\;m$, where $h_{c}$ is the canopy height) in the Speulderbos area of The Netherlands from August through December 1989. Concentrations ranged from < 0.1 μg m^−3^ to *>* 25 μg m^−3^, with the highest values measured during night time. Generally, a net deposition of ${NH}_{3}$ with a median value of 0.1 μg m^−2^ s^−1^ to the forest was observed with reversed concentration gradients observed occasionally. Measurements with oxalic acid denuders were made by Andersen et al. (1993) above a Norway spruce forest ($h_{c}=9\;m$) in the Ulborg Forest District in West Jutland, Denmark, during one week in spring (May 1991) and one week in late summer (August-September 1991). Out of a total of 80 sampling periods (3-hr duration), 34 had concentration differences between the two measurement heights statistically different from zero. Of these, 24 exhibited a positive gradient indicating deposition to the forest, while 10 had a negative gradient suggesting possible emission from the forest, although Andersen et al. (1993) suggest that 5 of these instances may have been due to advection from local sources. The mean ${NH}_{3}$ deposition flux (gradient method) measured over the periods was 0.02 μg m^−2^ s^−1^. Over six measurement periods from April 1988 to March 1990, ${NH}_{3}$ concentrations were obtained with oxalic acid denuders by Duyzer et al. (1994) at four heights (18, 22, 30 and 36 m above ground level (AGL)) above a Douglas fir forest ($h_{c}=15-18\;m$) in the Speulderbos area of The Netherlands. Estimated fluxes (gradient method) ranged from −1.27 μg m^−2^ s^−1^ (deposition) to 0.29 μg m^−2^ s^−1^ (emission), with only 10 % of all measured fluxes indicating potential emission from the forest. During the deposition periods, the authors asserted that deposition was enhanced when the canopy was wet or moist, but limited by stomatal uptake when the canopy was dry.

Denuder-based ${NH}_{3}$ concentrations and fluxes were again measured over the Douglas fir forest ($h_{c}=20\;m$) in Speulderbos, The Netherlands, by Wyers and Erisman (1998). Concentrations were measured at heights of 24.5, 28 and 34 m AGL and found to have a median concentration of 3.5 μg m^−3^, while observed fluxes (gradient method) were found to have distinct differences as a function of canopy wetness. When the canopy was very wet (after a recent rain or dewfall), ${NH}_{3}$ was efficiently deposited to the canopy, approaching the maximum flux possible via turbulent transport. On the other hand, when the canopy was dry, emissions were often observed upwards from the forest. In between, when the canopy was drying, deposition was less efficient than for a fully wet canopy and emissions were occasionally observed, possibly from evaporation of ${NH}_{3}$ dissolved in moisture on leaf surfaces.

Pryor et al. (2001) made above canopy (38 and 46 m AGL) measurements of ${NH}_{3}$ with wet effluent diffusion denuders (WEDD) at the Morgan-Monroe State Forest (MMSF) AmeriFlux tower in southern Indiana during four field campaigns - two spring periods (April-May 1998, 1999) and two winter periods (January 1999, February-March 2000). The MMSF tower is located in a primarily deciduous broadleaf forest (tulip poplar, white oak, red oak and sugar maple) with $h_{c}=25\;m$. During spring, concentrations ranged from 0.6 to 1.2 μg m^−3^, but substantially less (0.3 μg m^−3^) during winter. Most of the time in spring the forest acted as a sink for ${NH}_{3}$ with an average flux (gradient method) of 0.22 μg m^−2^ s^−1^. On some occasions (3 out of 17 measurement periods in April-May 1999), inverted concentration gradients were observed suggesting emission of ${NH}_{3}$ with an observed maximum upward flux of 0.6 μg m^−2^ s^−1^. Pryor et al. (2001) suggest that the upward fluxes may have been the result of either forest emission (from previously absorbed ${NH}_{3}$ evaporating as leaf surfaces dried) or evaporation of ammonium nitrate particles within the canopy.

Hansen et al. (2013) measured ${NH}_{3}$ concentrations (WEDD) and fluxes (relaxed eddy accumulation method; REA) during a 25-day period in the fall (October-November) over a beech forest ($h_{c}=26\;m$) at Lille Bøgeskov in Denmark. Leaf fall occurred during the measurement period and ${NH}_{3}$ fluxes were observed to change from deposition (0.25 μg m^−2^ s^−1^) before leaf fall to emission (up to a maximum of 0.67 μg m^−2^ s^−1^) after leaf fall. Hansen et al. (2013) hypothesized that the observed ${NH}_{3}$ emissions were the result of decaying litter from the fresh leaf fall or decreased canopy absorption of ${NH}_{3}$ (as a result of leaf fall) emitted by pre-existing litter or some combination of both processes. ${NH}_{3}$ fluxes were measured by Hansen et al. (2015) (again using WEDD and REA) during mid-summer (June, July, August 2013) and late-summer (September-October 2013) at the MMSF tower in southern Indiana. ${NH}_{3}$ fluxes were observed to be mostly upward (emission) with a maximum of 0.11 μg m^−2^ s^−1^. When ${NH}_{3}$ concentrations were larger or the canopy was wet, then deposition was observed with downward (deposition) fluxes up to 0.07 μg m^−2^ s^−1^.

In Hansen et al. (2017), the data of Hansen et al. (2013) from Denmark and of Hansen et al. (2015) from southern Indiana were re-analyzed with a two-layer, big-leaf bi-directional model (SURFATM-NH3, Personne et al., 2009) to investigate the partitioning of ${NH}_{3}$ fluxes between the ground surface, leaf cuticles and leaf stoma. The authors found that for the fully developed canopy of MMSF in summer, stomatal exchange dominated the daytime emissions of ${NH}_{3}$ (up to 0.05 μg m^−2^ s^−1^), while for the senescent canopy of Lille Bøgeskov, Denmark, the decomposing litter layer made the largest contribution to observed emissions from the forest (up to 0.15 μg m^−2^ s^−1^). The model was not able to account for the night time emissions observed at MMSF, but Hansen et al. (2017) hypothesized that these were due to cuticular desorption from the canopy.

More recently, Xu et al. (2023) measured ${NH}_{3}$ concentrations and calculated gradient-based fluxes using phosphoric-acid-impregnated filter packs mounted at multiple heights within and above a mixed deciduous forest at the Field Museum Tamakyuryo near Tokyo, Japan. Seasonal differences in concentrations and fluxes were observed, with higher values in summer and lowest in winter. Daytime fluxes during summer correlated strongly with solar radiation and exhibited upward (emissions) fluxes of 1–4 μg m^−2^ s^−1^, while nighttime fluxes were downward (deposition) and small (< 1 μg m^−2^ s^−1^). A bi-directional, big-leaf model applied to the data was able to adequately simulate daytime summer emissions, but was not able to reproduce the nighttime deposition, unless the cuticular resistance was modified to account for surface wetness.

It is clearly evident from this survey of prior flux measurements over forests that the environment of the forest canopy is complex and can result in either net deposition or emission of ${NH}_{3}$ depending on a variety of factors. In fact, Andersen and Hovmand (1999) present vertical profiles of ${NH}_{3}$ (their Fig. 4) within and above a forest in Lille Bøgeskov, Denmark, that suggest the canopy may act as a sink or source of ${NH}_{3}$ as function of height within the canopy. In their review paper, they present three profiles of ${NH}_{3}$ described as “with deposition” and one labelled “with emission”. The “with emission” profile exhibits a maximum near the forest floor and decreases with height through the canopy and becomes constant with height above the canopy. On the other hand, the profiles labeled “with deposition” have sharply decreasing gradients near canopy top as would be expected if ${NH}_{3}$ was being deposited rapidly. However, all three profiles increase again near the forest floor, suggesting that emission of ${NH}_{3}$ may be happening at or near the surface. In this exploratory study, we use a multi-layer canopy model to simulate these bi-directional interactions as a function of environmental conditions and height within the canopy for a mixed deciduous forest in western North Carolina. The questions we seek to explore include: (i) Can a multi-layer process model of ${NH}_{3}$ canopy exchange and vertical transport adequately reproduce the measurements observed in a mixed deciduous forest?; (ii) What physical and/or environmental processes contribute to the spatial and temporal nature of in-canopy ${NH}_{3}$ profiles and above-canopy hourly changes in concentrations?; and, (iii) Can different vertical extents of a mature forest simultaneously experience deposition and emission of ${NH}_{3}$ depending on the ambient air concentration and the effective vegetative compensation point? Answering these questions lie at the heart of a larger concern about whether a traditional “big-leaf” modelling approach is adequate to fully characterize ${NH}_{3}$ emission/deposition from/to a mature forest, a land-use type that covers a large fraction of the eastern United States.

## 2. Methods

### 2.1. Site description and measurements

Chemical concentration and flux measurements were made within and above the forest canopy from an aluminum walkup tower at the Coweeta Hydrologic Laboratory in western North Carolina, United States, over two years (2015 and 2016) as part of the Southern Appalachian Nitrogen Deposition Study (SANDS; Walker et al., 2023). The SANDS field campaign was designed to develop a reactive nitrogen deposition budget for a deciduous forest of the Southern Appalachians. The mixed deciduous forest ($h_{c}=35\;m$) surrounding the Coweeta tower (35.059 N, 83.427 W, 690 m ASL) is composed of black birch, tulip poplar, white oak, red maple, blackgum, sourwood and various hickory species (Oishi et al., 2018). The site also has a dense understory composed primarily of rosebay rhododendron, which is an evergreen shrub with maximum height of 4–7 m. Additional details of the Coweeta flux tower and site can be found in Oishi et al. (2018).

#### 2.1.1. Canopy physical measurements

**Fig. 1.**
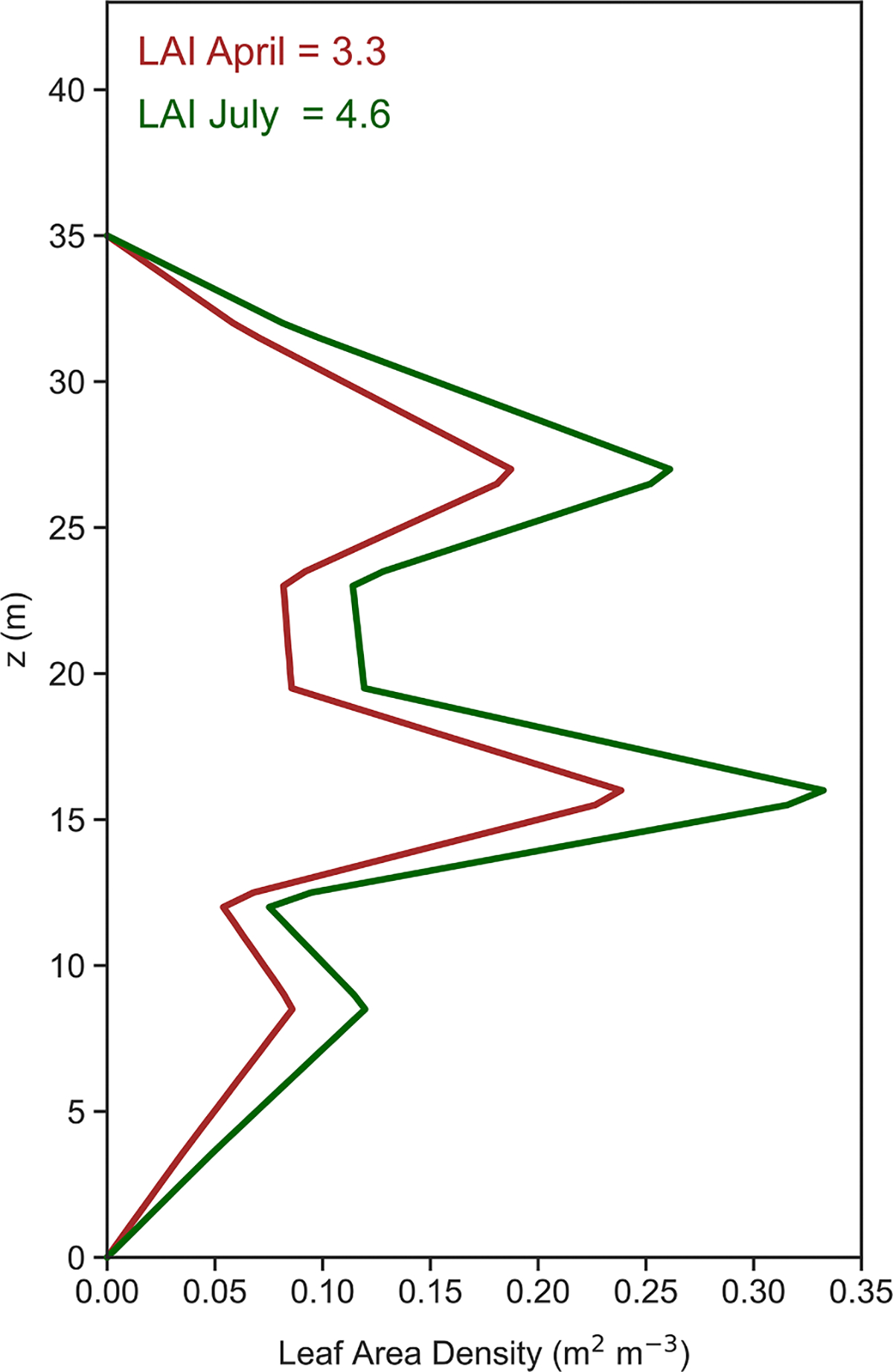
Vertical profile of leaf area density (LAD) within the Coweeta canopy as used in model simulations for April and July 2016.

The vertical leaf area profile was estimated from allometric measurements conducted during a previous study in which 87 trees of 10 species were sampled within the Coweeta basin (Martin et al., 1998). Measurements included diameter at breast height (DBH), total tree height, height from base of tree to base of live crown, stem and branch mass, leaf area and mass. The live crown was divided into thirds and branch and leaf metrics were calculated for each segment. To estimate the vertical leaf area profile, relationships between tree total height, height to base of live crown and leaf area to DBH were established for each species. Normalized cumulative leaf area (ranging from 0 to 1) was then related to crown segment (0 at base of live crown, 3 at top of live crown) as a sigmoid curve, allowing for estimation of a continuous (i.e. smooth) vertical leaf area profile. Individual curves were established for each species and for canopy position (upper canopy or subcanopy) as classified in the original data of Martin et al. (1998). These sets of equations were then applied to estimate vertical LAI profiles for deciduous trees from measurements of DBH collected in four 25 m × 25 m plots in the vicinity of the flux tower. The sampling of DBH and allometric estimates of maximum LAI by species within the tower plots are described by Oishi et al. (2018). The mean LAI profile in the four surrounding plots was then fit to the computational grid used in this study and a vertical leaf area density (LAD) profile was calculated as shown in Fig. 1. The measured peak summer canopy LAI of 4.6 was used for simulations in July 2016, while a value of 3.3 was used for April simulations, based on the LAI time series developed in Walker et al. (2023) (see Figure S6 and accompanying discussion in their Supplement).

#### 2.1.2. Meteorological measurements

Three-dimensional wind components along with momentum, sensible and latent heat, and carbon dioxide fluxes were measured above the canopy by eddy covariance (EC). The EC system comprised a closed path infrared gas analyzer (EC155, Campbell Scientific, Logan, UT) and sonic anemometer (Model 81000, R.M. Young Company, Traverse City, MI). Raw 10 Hz data were processed into hourly averages after block average detrending, 2D coordinate rotation and correction for spectral attenuation and density fluctuations (Novick et al., 2013). Site characteristics of micrometeorology and ecosystem fluxes of water and carbon dioxide have been previously described (Novick et al., 2013; 2014; Oishi et al., 2018). Air temperature and relative humidity were measured at the top of the tower (EC155, Campbell Scientific, Logan, UT) and 2/3 canopy height (HMP-45, Vaisala, Helsinki, Finland). Photosynthetically active radiation (PAR; LI-190, LI-COR Biosciences, Lincoln, NE) and upward and downward, shortwave and longwave radiation (CNR 4, Kipp & Zonen, Delft, The Netherlands) were measured at the top of the tower. Surface wetness was measured in the canopy crown (32 m), understory (10.5 m) and at the ground using leaf wetness sensors (Model 237, Campbell Scientific). Soil volumetric water content (VWC) averaged over 0–30 cm depth was measured in four locations around the tower using time domain reflectometry probes (CS616, Campbell Scientific). Soil temp was measured at four depths (5, 20, 35, and 55 cm) in two locations near the tower using thermistors. For missing data, linear interpolation was used to fill short gaps (1–4 h). Longer gaps were filled by substitution using the average hourly diel profile calculated for each month.

#### 2.1.3. Air concentration measurements

Concentrations of ${NH}_{3},\;{HNO}_{3},\;{SO}_{2},\;{NH}_{4}^{+},\;{NO}_{3}^{-}$, and ${SO}_{4}^{2-}$ in air were measured concurrently at 10 heights from just above the forest floor (0.5 m AGL) to several meters above the canopy (37.5 m during spring 2016, 43.5 m during summer, 2016) using a glass annular denuder/filter pack (URG Corporation, Chapel Hill, NC) system. The sampling assembly included a 1 % Na_2_CO_3_ coated denuder for collection of acid gases followed by a 1 % H_3_PO_3_ coated denuder for collection of ${NH}_{3}$, and a filter pack containing a primary Teflon filter for collection of aerosol and a backup Nylon filter (47 mm, Pall Corp, Port Washington, NY) to collect ${HNO}_{3}$ liberated by dissociation of NH_4_NO_3_ on the primary filter. Inlets were Teflon coated glass impactors with a nominal 2.5 μm aerodynamic diameter cutpoint (URG Corporation, Chapel Hill, NC). Sample durations were typically 3 or 4 h at a flow rate of ~ 16.7 L min^−1^. Flow rates were controlled by critical orifice and were checked before and after each sampling period with a NIST traceable primary standard flow meter (Bios DryCal DC-Lite flowmeter, Mesa Laboratories, Inc., Lakewood, CO).

Denuders and filters were extracted with 10 mL of deionized water and analyzed by ion chromatography (IC, Dionex model ICS-2100, Thermo Scientific, Waltham, MA). Extracts were analyzed for cations using Dionex IonPac 2 mm CG12 guard and CS12 analytical column**s**; separations were conducted using 20 mM methanesulfonic acid (MSA) as eluent at a flow rate of 0.25 mL/min. Anions were analyzed (IonPac 2 mm AG23 guard column, AS23 analytical columns) using an isocratic eluent mix of carbonate/bicarbonate (4.5/0.8 mM) at a flow rate of 0.25 mL/min. Multi-point (≥5) calibrations were conducted using a mixture prepared from individual inorganic standards (Inorganic Ventures, Christiansburg, VA). A mid-level accuracy check standard was prepared from certified standards mix (AccuStandard, New Haven, CT) for quality assurance/quality control. Throughout the field campaign, duplicate samples were taken at two locations on the tower, at the lowest (0.5 m AGL) and highest (37.5 m in spring and 43.5 m in summer) measurement levels. Measurement uncertainty was calculated as the relative deviation from the mean: $\left(C_{i}-C_{\mathrm{mean}}\right)/C_{\mathrm{mean}}\times 100\%$, where $C_{i}$ is concentration of the duplicate sample #1 or #2 and $C_{\mathrm{mean}}$ is the mean concentration of the two duplicate samples. Mean and median relative errors for ${NH}_{3}-\text{N}$ were 9.1 and 5.7 %, respectively.

Hourly concentrations of ${NH}_{3}$ were measured at two heights above the canopy (34m and 37.5 m during spring, 2016; 34 m and 43.5 m during summer, 2016) using the Monitor for Aerosols and Gases in Ambient Air (MARGA, Metrohm Applikon B.V., the Netherlands). Details and principles of the MARGA system have been previously described (Rumsey and Walker, 2016; Chen et al., 2017). Briefly, the MARGA 2S consisted of two sampler boxes positioned on the tower and a detector box located in a climate-controlled enclosure at the base of the tower. Sample boxes comprised a 30 cm long PFA Teflon inlet with no particle size selection, through which air flow was mass controlled at ~ 16.7 L min^−1^, a wet rotating denuder (WRD) for collection of soluble gases and a steam jet aerosol collector (SJAC). Liquid sample from the WRD and SJAC is continuously drawn from the sample boxes down the tower to the analytical box for analysis by Ion Chromatography (IC) on an hourly basis at the detector unit located in a climate-controlled enclosure at the base of the tower. At the beginning and end of the measurement intensive, liquid ${NH}_{4}^{+}$ standards were introduced at the WRD and SJAC, with airflow turned off, to assess accuracy of the ${NH}_{3}$ measurement.

#### 2.1.4. Biogeochemical measurements

Ammonia emission potentials and compensation points for live vegetation, leaf litter, and soil were estimated from measurements of ${NH}_{4}^{+}$ and pH in the leaf tissue and soil pore water. Green leaves were collected from 18 species within the flux footprint of the tower and other locations in the Coweeta basin. Leaf litter was collected along transects extending to the northeast and southwest (i.e., predominant wind directions) of the flux tower approximately 100 m. Soil chemistry was measured in 20 m × 20 m plots located in the vicinity of the tower. During 2010, soil ${NH}_{4}^{+}$ was determined on soil samples collected with PVC cores (5 cm diameter and 10 cm deep) in four locations (replicates) within each of four plots. Samples were collected bi-monthly during the growing season. Soil emission potential was estimated directly from measured molar concentrations of [$H^{+}$] and [${NH}_{4}^{+}$]. Estimated emission potentials varied widely for green leaves, senescent leaves and litter (see Table S9 in Walker et al.; 2023). Sensitivity runs of the model indicated that results were most sensitive to the litter emission potential and less so for the stomatal emission potential. Values for the simulations presented here fall well within the ranges presented in Table S9 of Walker et al. (2023). Additional details of the emission potential measurements can be obtained from Walker et al. (2023).

### 2.2. Model description

The Atmospheric Chemistry and Canopy Exchange Simulation System (ACCESS) is a multi-layer modeling system that can be used to simulate one-dimensional atmospheric chemistry, vertical turbulent transport and biosphere-atmosphere exchange processes from the surface through a user-defined upper boundary (Saylor, 2013). ACCESS-NH3 is a separate version of the modeling system designed specifically to simulate the bi-directional exchange of ${NH}_{3}$ between the surface (including soils, plant litter and an overlying canopy) and the atmosphere. Application of ACCESS-NH3 to a particular simulation application is accomplished via a combination of a simulation control file, input data files and peripheral tools. The governing equation of ACCESS-NH3 is given by

(1) $$\frac{\partial \chi (z,\;t)}{\partial t}=-\frac{\partial F(z,\;t)}{\partial z}+S(z,t)$$

where,

χ(z,t)= concentration of ${NH}_{3}$ (mol cm^−3^);

F(z,t)= vertical turbulent flux of ${NH}_{3}$ (mol cm^−2^ s^−1^); and,

S(z,t)= source/sink flux of ${NH}_{3}$ from/to the plant canopy (mol cm^−3^ s^−1^).

On application of Reynolds turbulent decomposition and time averaging, the vertical turbulent flux can be expressed as

(2) $$F(z,t)=\bar{w^{\prime }\chi^{\prime }}(z,t)$$

where,

$w^{\prime }=$ fluctuation of the vertical velocity (cm s^−1^);

$\chi^{\prime }=$ fluctuation of the ${NH}_{3}$ concentration (mol cm^−3^); and,

the overbar represents time averaging.

Using gradient diffusion theory, the vertical turbulent flux is approximated as

(3) $$\bar{w^{\prime }\chi^{\prime }}(z,t)=-\rho (z,t)K_{v}(z,t)\frac{\partial \chi (z,\;t)/\rho (z,\;t)}{\partial z}$$

where,

$K_{\nu }(z,t)=$ scalar turbulent eddy diffusivity (cm^2^ s^−1^); and,

ρ(z,t)= molar air density (molcm^−3^).

Eq. (1) then becomes

(4) $$\frac{\partial \chi (z,t)}{\partial t}=\frac{\partial }{\partial z}\left(\rho (z,t)K_{v}(z,t)\frac{\partial \chi (z,\;t)/\rho (z,\;t)}{\partial z}\right)+S(z,\;t)$$

**Fig. 2.**
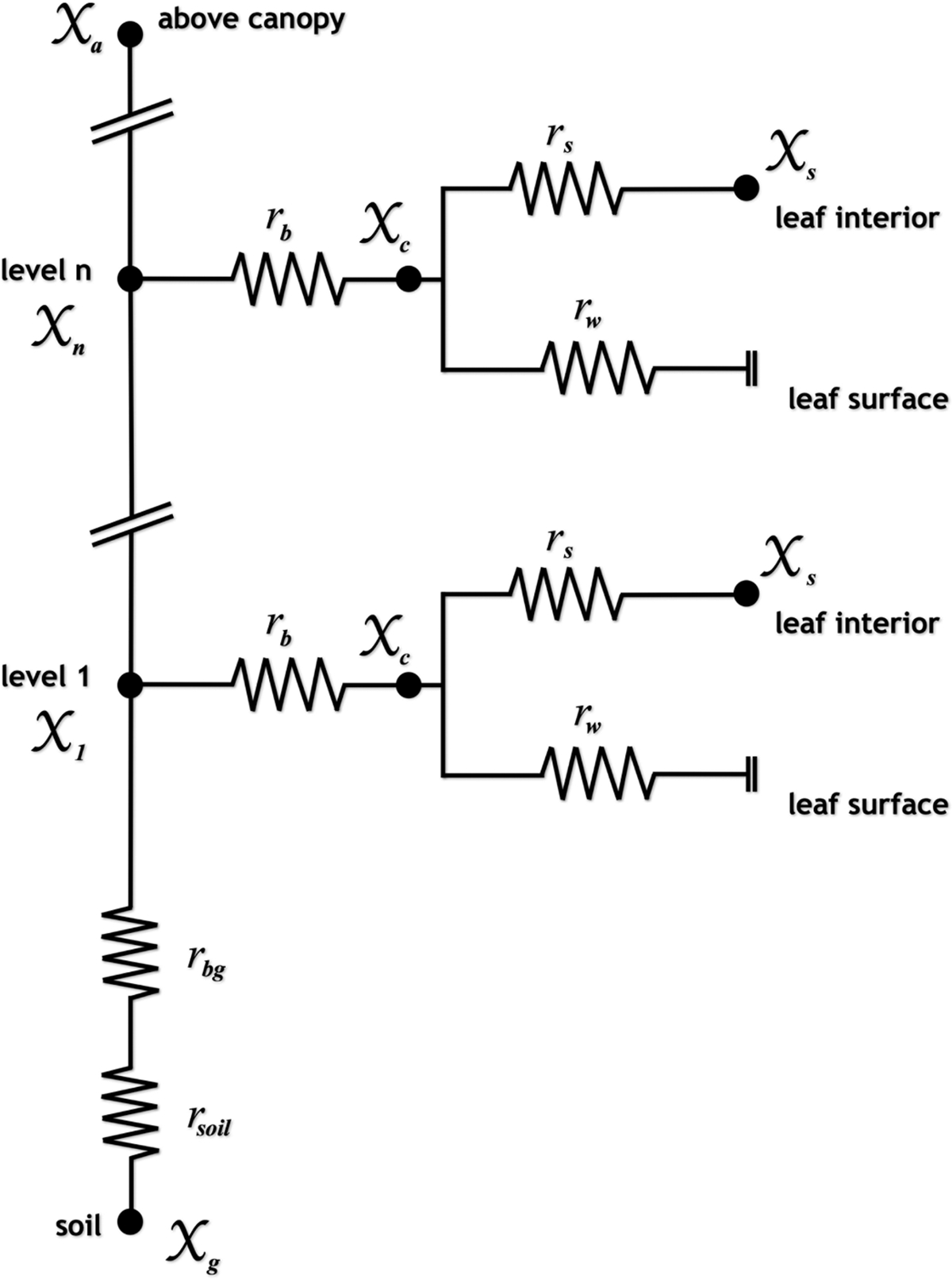
Resistance formulation for the multilayer ACCESS-NH3 canopy model (see text for symbol definitions).

At each level in the canopy, exchange of ${NH}_{3}$ with the canopy is parameterized with a resistance analogy approach as illustrated in Fig. 2. An NH_3_ molecule diffuses from the ambient air at a level n within the canopy and encounters a resistance to diffusion across the quasi-laminar boundary layer adjacent to the leaf surface, $r_{b}$. Once it has diffused through the boundary layer, the ${NH}_{3}$ molecule may then diffuse into a leaf stoma and be absorbed into the plant’s apoplast or it may deposit onto the cuticle of the leaf surface. Because NH_3_ is naturally present in the interior of leaves (as ammonium, ${NH}_{4}^{+}$), if the apoplastic ${NH}_{4}^{+}$ concentration is high enough, ${NH}_{3}$ will desorb and diffuse out of the stoma and across the boundary layer into the ambient air. In this way, vegetation may serve as either a source or a sink for gaseous ${NH}_{3}$ depending on the concentration difference between the canopy air and the sub-stomatal cavity, and the source or sink nature of the canopy may be a function of height above the ground surface. The gas-phase ${NH}_{3}$ concentration in the plant’s sub-stomatal cavities that is in equilibrium with the apoplastic ${NH}_{4}^{+}$ concentration is referred to as the stomatal compensation point, $\chi_{s}$. If an effective overall canopy compensation point, $\chi_{c}$, is defined, then component fluxes of ${NH}_{3}$ can be parameterized as

(5) $$F_{c}(z,\;t)=-\frac{\chi (z,\;t)-\chi_{c}(z,\;t)}{r_{b}(z,\;t)}$$

(6) $$F_{s}(z,\;t)=-\frac{\chi_{c}(z,\;t)-\chi_{s}(z,\;t)}{r_{s}(z,\;t)}$$

(7) $$F_{w}(z,\;t)=-\frac{\chi_{c}(z,\;t)}{r_{w}(z,\;t)}$$

where,

$F_{c}(z,\;t)=$ total flux of ${NH}_{3}$ between the canopy and the ambient air (mol cm^−2^ s^−1^);

$F_{s}(z,\;t)=$ flux of ${NH}_{3}$ between leaf stoma and the ambient air (mol cm^−2^ s^−1^);

$F_{w}(z,\;t)=$ flux of ${NH}_{3}$ between cuticular leaf surfaces and the ambient air (mol cm^−2^ s^−1^);

$\chi_{c}(z,\;t)=$ total canopy effective ${NH}_{3}$ compensation point (mol cm^−3^);

$\chi_{s}(z,\;t)=$ stomatal ${NH}_{3}$ compensation point (mol cm^−3^);

$r_{b}(z,\;t)=$ resistance to diffusion of ${NH}_{3}$ through the quasi-laminar boundary layer on the leaf surface (s cm^−1^);

$r_{s}(z,\;t)=$ resistance to uptake of ${NH}_{3}$ into leaf stoma (s cm^−1^); and,

$r_{w}(z,\;t)=$ resistance to uptake of ${NH}_{3}$ onto leaf cuticles (s cm^−1^).

The total canopy flux of ${NH}_{3}$ can also be expressed as just the sum of the stomatal and cuticular fluxes

(8) $$F_{c}(z,\;t)=F_{s}(z,\;t)+F_{w}(z,\;t).$$

The source function, S(z,t), is then defined as

(9) $$S(z,\;t)=F_{c}(z,\;t)\cdot a(z)$$

or,

(10) $$S(z,\;t)=-v_{c}(z,\;t)\left(\chi (z,\;t)-\chi_{c}(z,\;t)\right)\cdot a(z)$$

With

$v_{c}(z,\;t)=$ exchange coefficient of ${NH}_{3}$ between the canopy and the ambient air (cm s^−1^); and,

a(z)= leaf area density profile of the canopy (cm^2^ cm^−3^).

The total canopy effective compensation point can be determined by combining Eqs. (5–8) and solving for $\chi_{c}$ to get (dropping the (z,t) notation for convenience)

(11) $$\chi_{c}=\frac{r_{s}r_{w}\chi \;+\;r_{b}r_{w}\chi_{s}}{r_{s}r_{w}\;+\;r_{b}r_{w}\;+\;r_{b}r_{s}}.$$

The final governing equation of ACCESS-NH3 is then given by

(12) $$\frac{\partial \chi (z,\;t)}{\partial t}=\frac{\partial }{\partial z}\left(\rho (z,\;t)K_{v}(z,\;t)\frac{\partial \chi (z,\;t)/\rho (z,\;t)}{\partial z}\right)-v_{c}\left(z,\;t\right)\left(\chi \left(z,\;t\right)-\chi_{c}\left(z,\;t\right)\right)a\left(z\right).$$

An initial concentration profile is provided at the beginning of a simulation as

(13) $$\chi (z,0)=\chi_{0}(z).$$

At the top of the domain (z=H), measured ${NH}_{3}$ concentrations are provided as the upper boundary condition as

(14) $$\chi (H,t)=\chi_{a}(t),$$

while at the ground surface (z=0), the ${NH}_{3}$ flux is parameterized as an exchange between the ambient air next to the surface and an ${NH}_{3}$ compensation point in the soil and/or litter at the surface

(15) $$-\rho (0,\;t)K_{v}(0,\;t)\frac{\partial \chi (0,\;t)/\rho (0,\;t)}{\partial z}=-v_{s}(t)\left(\chi (0,\;t)-\chi_{g}(t)\right),$$

where,

$v_{s}(t)=$ exchange coefficient between the soil/litter and the atmosphere (cm s^−1^);

$\chi_{0}(z)=$ initial ${NH}_{3}$ concentration profile (mol cm^−3^);

$\chi_{g}(t)=$ soil compensation point for ${NH}_{3}$ (mol cm^−3^); and,

H= top of the computational domain (cm).

Canopy physics parameterizations included in the model are presented fully in Supplement I, but are briefly described here. Within the canopy, the turbulent eddy diffusivity profile is computed with a formulation derived from Raupach (1989). Above the canopy, the surface layer parameterization of Stull (1988) and Seinfeld and Pandis (1998) is used, including stability corrections, and ensuring continuity between within- and above-canopy eddy diffusivities. The leaf area density (LAD) profile, the volumetric density of leaf area as a function of vertical extent, is provided in an input file for the particular canopy being simulated. Radiative fluxes through the computational domain are determined via the algorithms of Bodin and Franklin (2012) with attenuation of the photosynthetic photon flux density (PPFD) and near-infrared (NIR) components calculated as a function of LAD in both sunlit and shaded portions of the canopy, while longwave (LW) radiation fluxes are calculated after Norman (1979). Using the computed radiation components, a leaf energy balance is formulated at each level for the sunlit and shaded fractions of the canopy and a separate leaf temperature is determined for each. The photosynthetic assimilation rate in each fraction at each canopy level is calculated from the Campbell and Norman (1998) formulation as derived from Collatz et al. (1991), with parameters as suggested by Houborg et al. (2009) for a temperate deciduous forest. The standard formulation of Hicks et al. (1987) is used for the leaf quasi-laminar boundary layer resistance, but is modified to account for the reduced wind speed (and hence greater resistance) as a function of depth within the canopy. The leaf stomatal resistance is calculated using the formulation of Medlyn et al. (2011) with parameters appropriate for a mixed deciduous forest. The formulation of Flechard et al. (2010) is used for the NH_3_-specific leaf cuticular resistance, which is a function of relative humidity and leaf temperature. The mean wind speed within the canopy is computed from the empirical relation as described by Meyers et al. (1998) with parameters set appropriate for a forest. Exchange of ${NH}_{3}$ with soil/litter at the surface is parameterized with a two resistances in series approach to represent transport across the litter layer and a quasi-laminar boundary layer near the surface. And finally, profiles of air temperature and humidity throughout the computational domain are deterministically simulated via coupled energy and mass balance equations adapted from Bonan et al. (2018). Further details of the canopy physics formulations can be found in Supplement I to this manuscript.

**Fig. 3.**
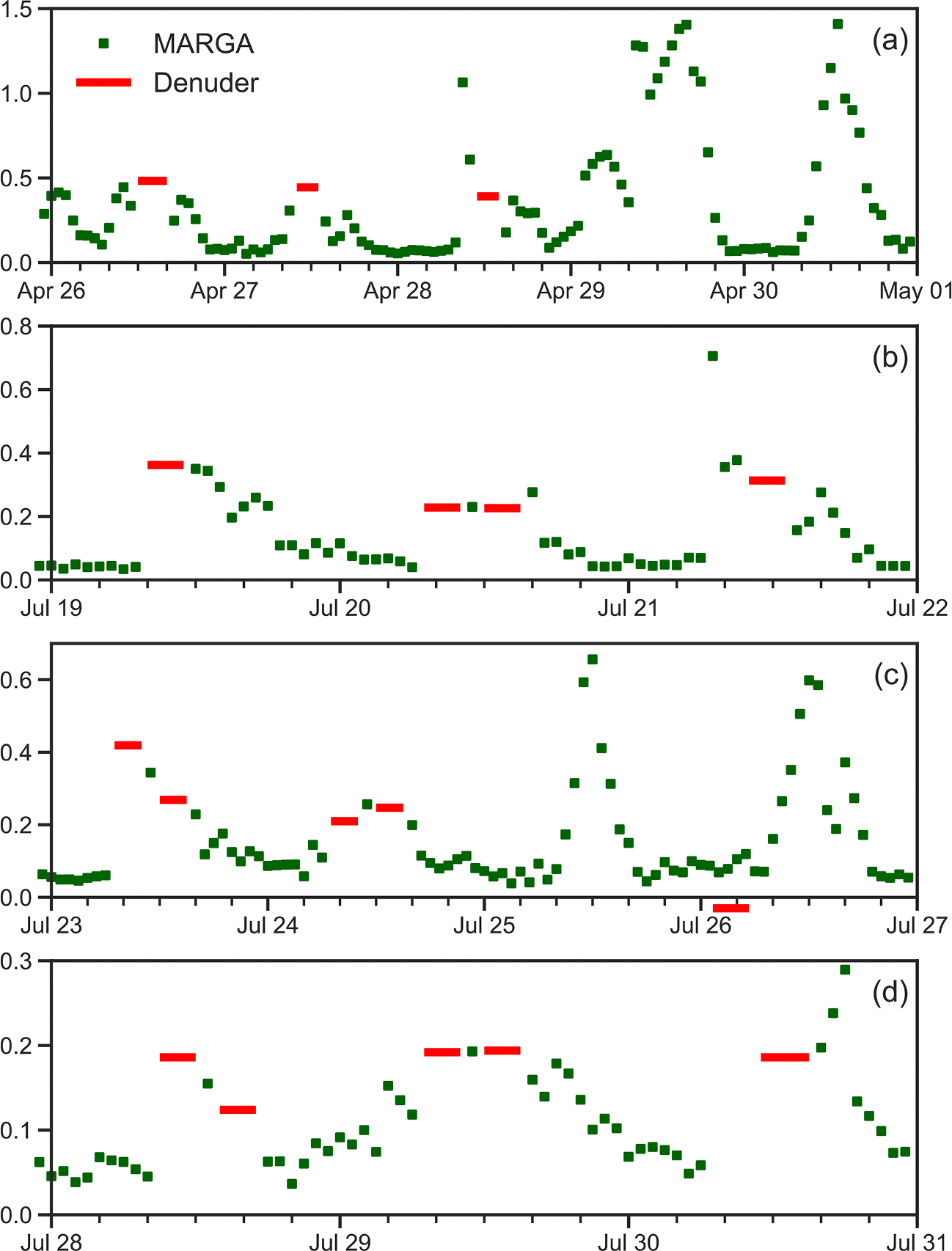
Measured ${NH}_{3}$ concentrations (μg m^−3^) from denuders (red) and adjusted MARGA averages (green) which are used as top-of-domain boundary conditions in ACCESS-NH3 simulations.

Inputs to ACCESS-NH3 for the Coweeta measurement site include site location as latitude-longitude, simulation start time, canopy height and LAD profile, soil type, topsoil depth, soil heat flux, and meteorological data measured above the canopy (air temperature, pressure, relative humidity, mean wind speed, friction velocity, downwelling shortwave radiation, PPFD, latent heat flux and sensible heat flux). Emission potentials for ${NH}_{3}$ are also input to the modeling system and the values chosen were guided by measurements in the soil/litter and in the canopy (Walker et al., 2023). An important simulation input is specification of the ${NH}_{3}$ boundary condition at the top of the computational domain (Eq. (14)). The MARGA instrument provided continuous ${NH}_{3}$ concentration measurements over each period and served as the basis for the upper boundary condition in model simulations. Because of inherent uncertainties in the magnitude of concentration values obtained from the MARGA during the study period, the coincident ${NH}_{3}$ denuder measurements have been used to adjust the MARGA values to be more consistent with multi-hour averaged ${NH}_{3}$ concentrations as measured by the denuders. ${NH}_{3}$ concentration boundary conditions at the domain top for each of the simulation periods are shown in Fig. 3, where denuder values (shown in red) were used when those measurements were available and adjusted MARGA values (shown in green) were used otherwise.

## 3. Results and discussion

**Fig. 4.**
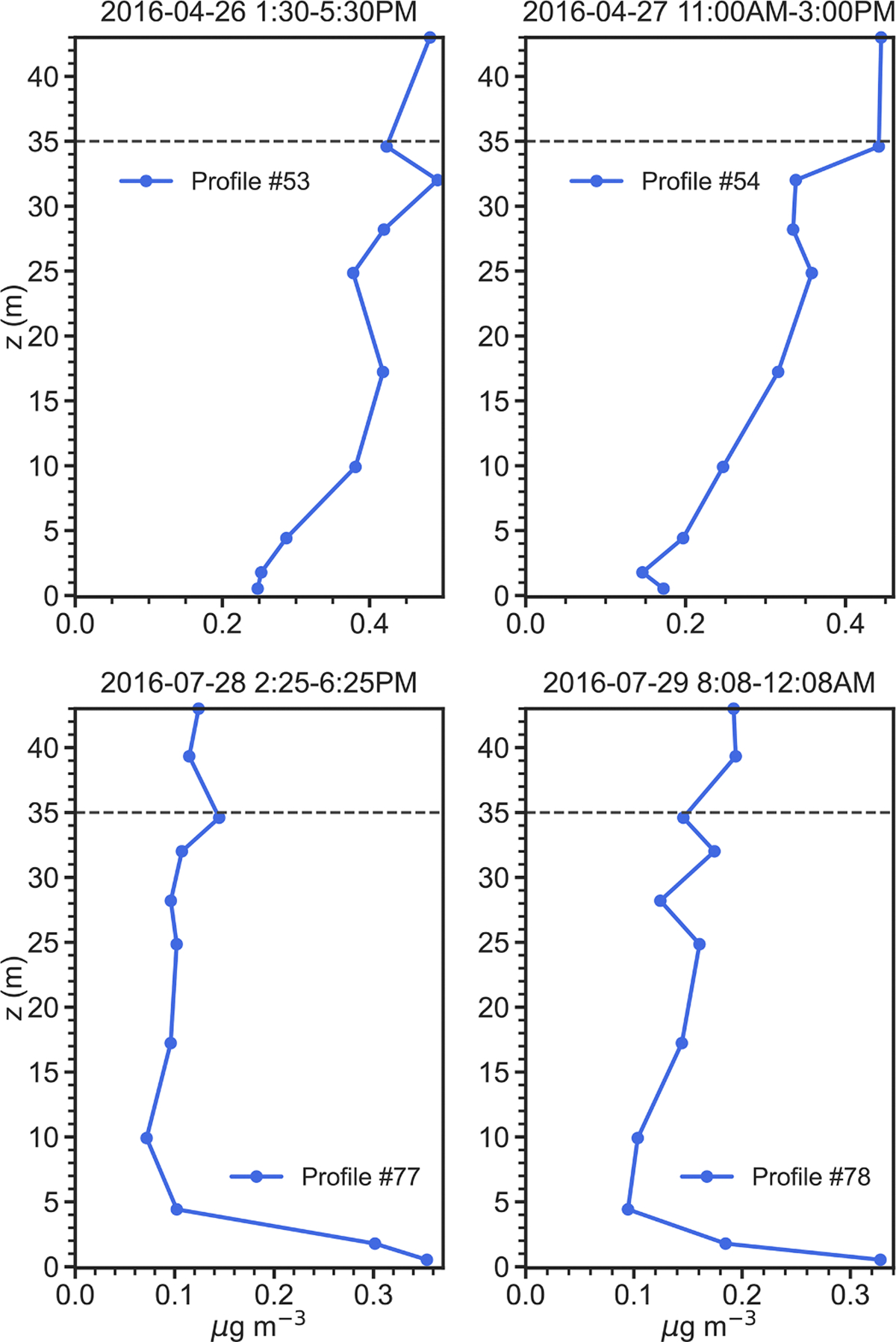
Denuder-based ${NH}_{3}$ profiles (μg m^−3^) in April (#53 and #54) compared with profiles in July (#77 and #78). Horizontal dashed line indicates canopy top.

Four simulation periods during the 2016 intensive were chosen for analysis (Table 1): (i) April 26–30; (ii) July 19–21; (iii) July 23–26; and, (iv) July 28–30. One reason for selecting these periods was that as shown in Fig. 4, measured ${NH}_{3}$ profiles within the canopy exhibited a very different nature between April and July. Profiles for April (53 and 54 in Fig. 4), display generally decreasing concentrations from canopy top down to the forest floor. In contrast, profiles for July (77 and 78 as examples in Fig. 4) present constant or slightly decreasing concentrations from canopy top to just above the forest floor, where a sharp increase occurs in the lowest 5 m. Understanding the reasons for these profile differences between April and July was a prime motivation for this work. A second reason for choosing these specific periods lay in the availability of continuous micrometeorological and chemical data to drive ACCESS-NH3 simulations for these times.

The simulation domain was defined differently for the April and July periods, with the computational grid extending from the forest floor to a height of 37 m AGL in April and 43 m AGL July, near where the topmost chemical and micrometeorological measurements were obtained during each period. A vertical grid resolution of 0.5 m was used, resulting in a total of 75 grid nodes for the April simulations and 87 grid nodes for July. Based on the LAI measurements described in Section 2.1, a leaf area density profile was defined on each computational grid, approximating Fig. 1, with a canopy height of 35 m and an overall LAI of 3.3 for April simulations and 4.6 for July simulations. Simulations were driven by micrometeorological data averaged over 30 min, with both boundary concentrations and environmental data kept constant over each 30-minute simulation period even though the integration time step for the simulation was nominally 5 s or less.

**Fig. 5.**
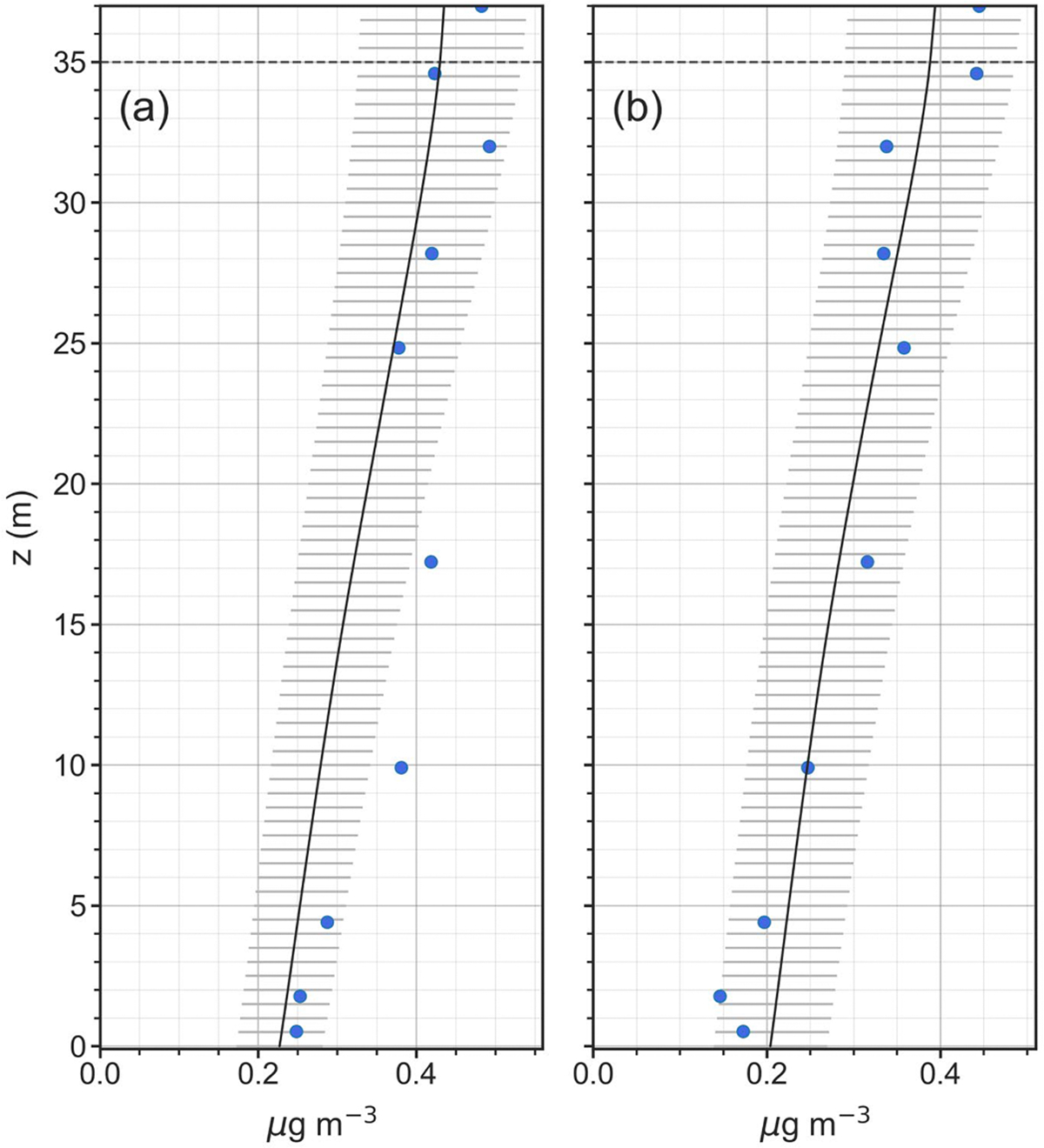
Simulation results for (a) Profile #53 on April 26, 2016, 1:30–5:30PM, and (b) Profile #54 on April 27, 2016, 11:00AM – 3:00PM. In each panel, the mean modeled ${NH}_{3}$ concentration (μg m^−3^) profile (solid black line) is shown with ± 1 standard deviation (horizontal gray lines) and denuder-based NH_3_ observations (μg m^−3^) shown as blue symbols. Horizontal dashed gray line denotes canopy top.

Fig. 5 presents modelled concentration profiles for April 26 and 27. Volumetric source/sink profiles (i.e., S(z,t) in Eq. (1)) for these same profiles are presented in Figure S-1 in Supplement II. In these figures, model results are averaged over the same time period during which denuder profile measurements (in blue) were obtained (Profile #53 on April 26 and Profile #54 on April 27), with the solid black line as mean values and the horizontal gray lines at each model level showing ± one standard deviation of the model results over the time period. Modelled canopy profiles of stomatal flux ($F_{s}$), cuticular flux ($F_{w}$) and total canopy flux ($F_{c}$) are shown in Figures S-2 and S-3 of Supplement II, also with means and standard deviations over the measurement time periods. For these two periods in April, the model is able to adequately replicate the denuder observations with decreasing ${NH}_{3}$ concentrations occurring from the top of the canopy down to the forest floor. The volumetric source/sink profiles (Figure S-1) exhibit deposition all throughout the canopy, while the component canopy flux profiles (Figures S-2 and S-3) show deposition to leaf surfaces ($F_{w}$) larger in the understory and lower canopy but stomatal deposition ($F_{s}$) dominating in the upper canopy (where ${NH}_{3}$ concentrations are larger).

**Fig. 6.**
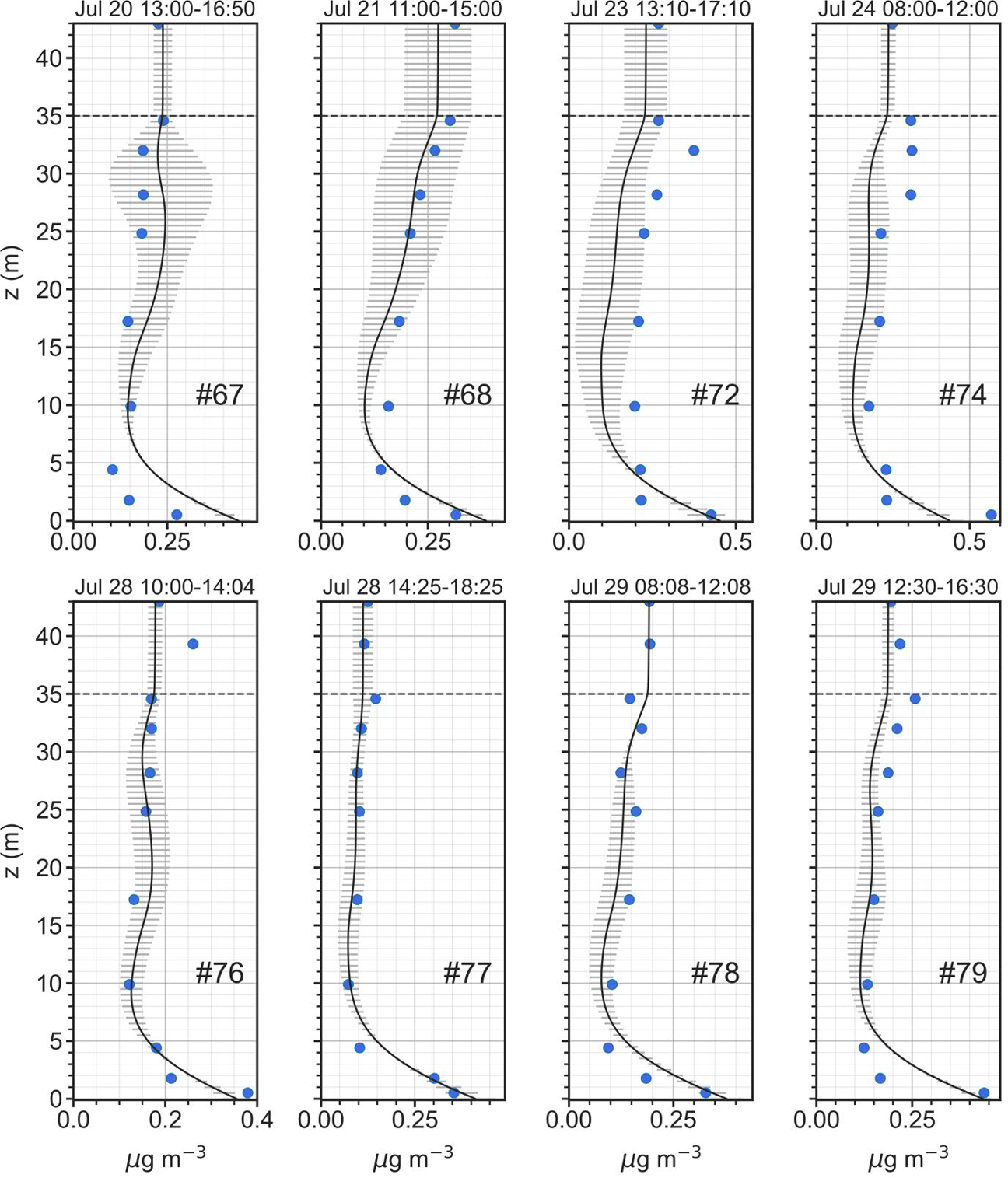
Simulation results for Profiles 67, 68, 72, 74, 76, 77, 78 and 79 taken during July 2016. Mean modeled ${NH}_{3}$ concentration (μg m^−3^) profiles (solid black lines) are shown with ± 1 standard deviation (horizontal gray lines) and denuder-based ${NH}_{3}$ observations (μg m^−3^) given as blue symbols. Horizontal dashed gray line denotes canopy top.

**Fig. 7.**
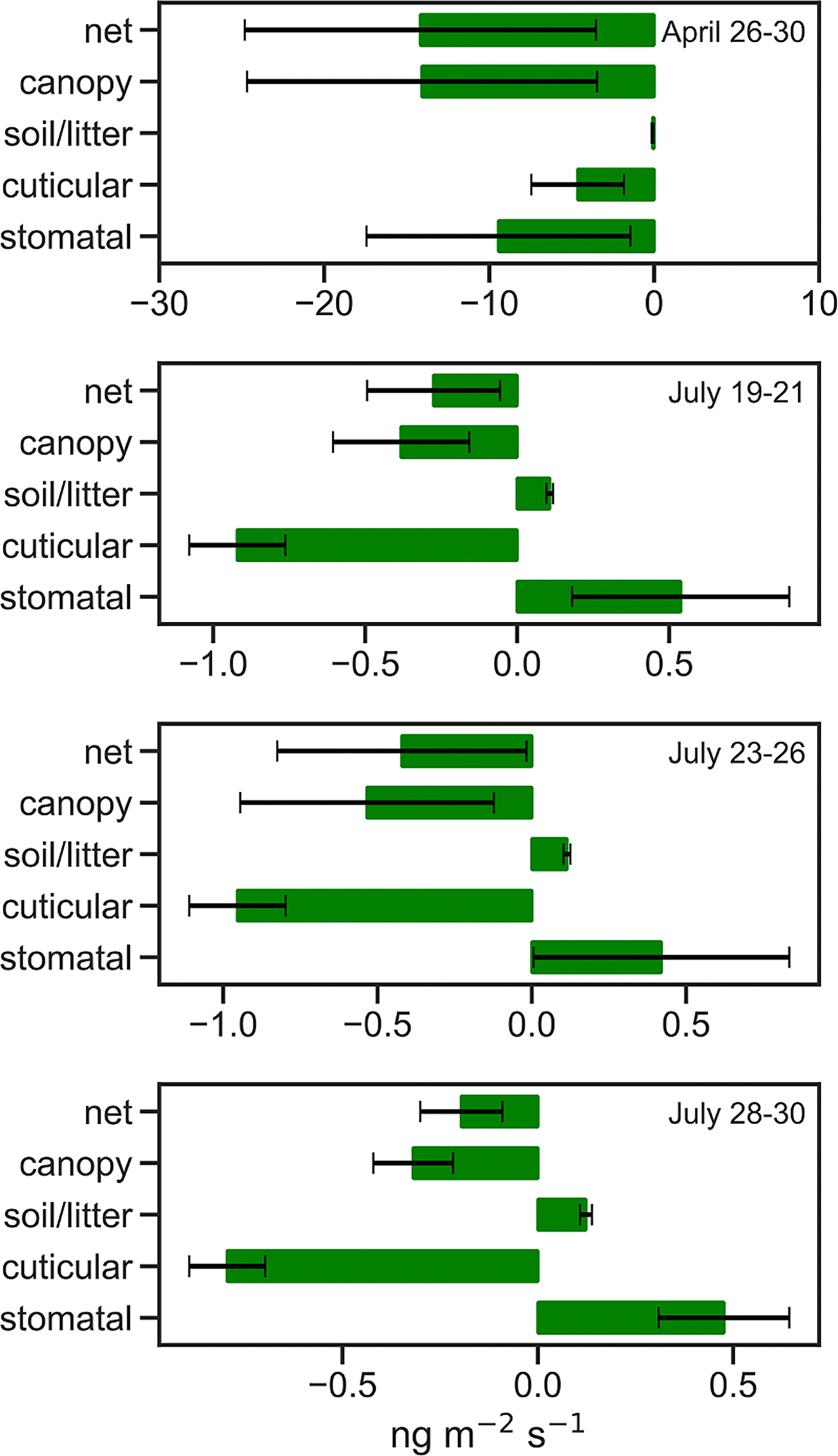
Mid-day (10:00am – 2:00pm) component fluxes integrated over the entire domain for each simulation period.

Model concentration profile results and denuder observations for selected July measurement periods (Profiles 67, 68, 72, 74, 76, 77, 78 and 79) are shown in Fig. 6. For these periods the model does a reasonably good job of replicating the denuder profiles, with constant or slightly decreasing concentrations from canopy top to just above the forest floor. In the lowest 3–5 m, ${NH}_{3}$ concentrations increase dramatically, as a result of a source at ground level (from vegetative litter on the forest floor). For these periods, the volumetric source/sink profiles (Figure S-4 in Supplement II) generally exhibit deposition in the lower canopy and upper canopy, but emission in the mid-canopy area. Canopy stomatal and cuticular fluxes for these profiles (Figures S-5 through S-12) show stomatal emissions in the mid-canopy that are partially offset by deposition to leaf surfaces throughout the entire canopy. Stomatal emissions in the mid-canopy region occur as a result of the effective canopy compensation point being larger than the ${NH}_{3}$ concentration in air at that region. Integrated over the entire canopy, as seen in Fig. 7, for the July measurement periods, the model suggests that stomatal and soil/litter emissions are countered by deposition to leaf surfaces, resulting in a small net deposition for the entire system. On the other hand, for the April time period, there is deposition via the stomata, to the forest floor and to leaf surfaces. This finding is consistent with the source-sink inverse modelling results at the same site by Wu et al. (2023).

**Fig. 8.**
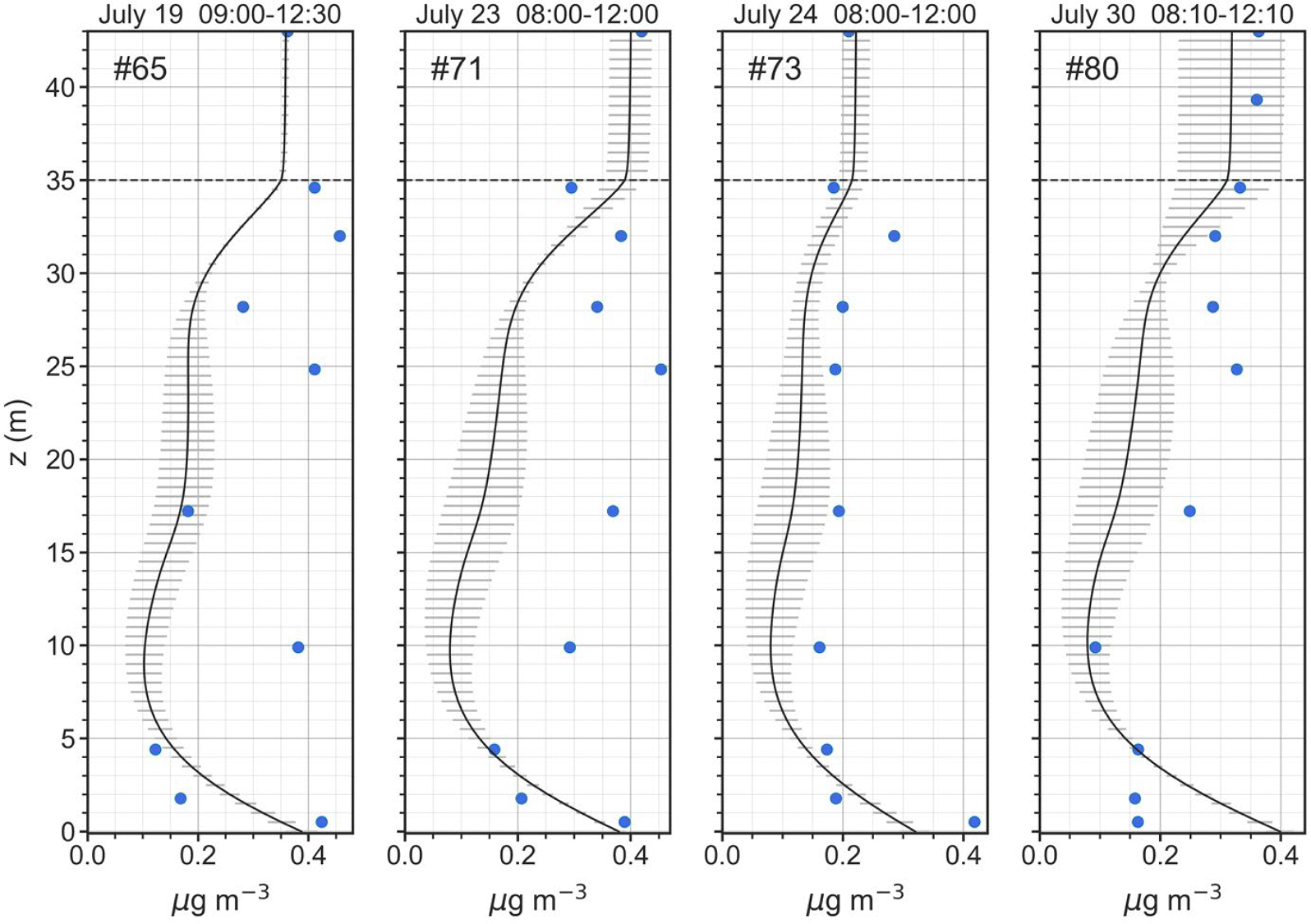
Mean modeled ${NH}_{3}$ concentration (μg m^−3^) profile (solid black line) with ± 1 standard deviation (horizontal gray lines) and denuder-based ${NH}_{3}$ observations (μg m^−3^) as blue symbols for Profile #65, 9:00AM – 12:30PM on July 19; Profile #71, 8:00AM – 12:00PM on July 23, 2016; Profile #73, 8:00AM – 12:00PM on July 24, 2016; and, Profile #80, 8:10AM – 12:10PM on July 30, 2016.

**Fig. 9.**
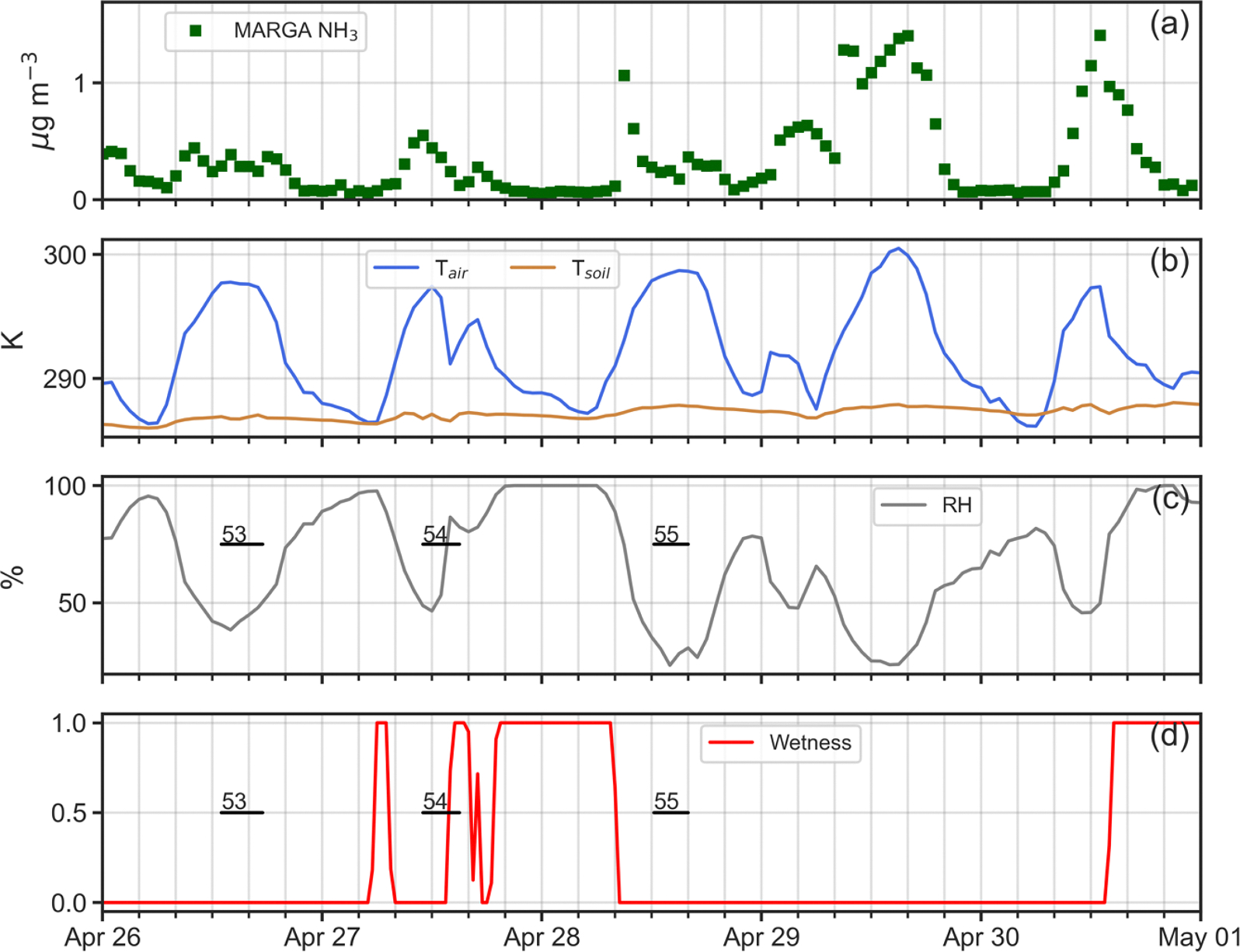
Time series plots of (a) adjusted MARGA ${NH}_{3}$ concentrations (μg m^−3^); (b) in-canopy (20 m AGL) air temperature (K) and 5 cm soil temperature (K); (c) in-canopy (20 m AGL) relative humidity (%); and, (c) canopy wetness index at 32 m AGL (0–1) for April 26–30, 2016. Temporal extent of denuder-based ${NH}_{3}$ profiles 53, 54 and 55 shown as horizontal black lines.

**Fig. 10.**
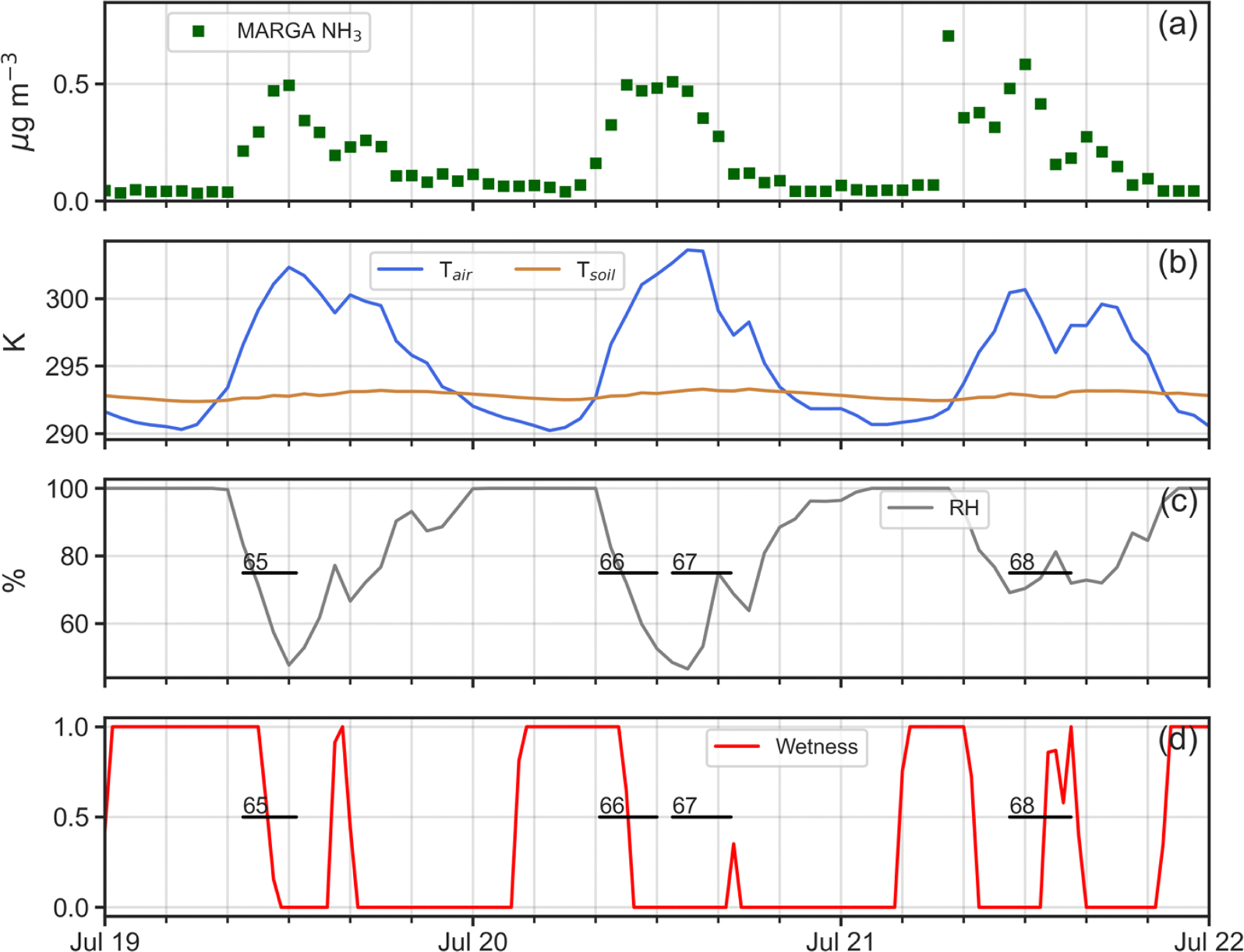
Time series plots of (a) adjusted MARGA ${NH}_{3}$ concentrations (μg m^−3^); (b) in-canopy (20 m AGL) air temperature (K) and 5 cm soil temperature (K); (c) in-canopy (20 m AGL) relative humidity (%); and, (c) canopy wetness index at 32 m AGL (0–1) for July 19–21, 2016. Temporal extent of denuder-based ${NH}_{3}$ profiles 65, 66, 67 and 68 shown as horizontal black lines.

**Fig. 11.**
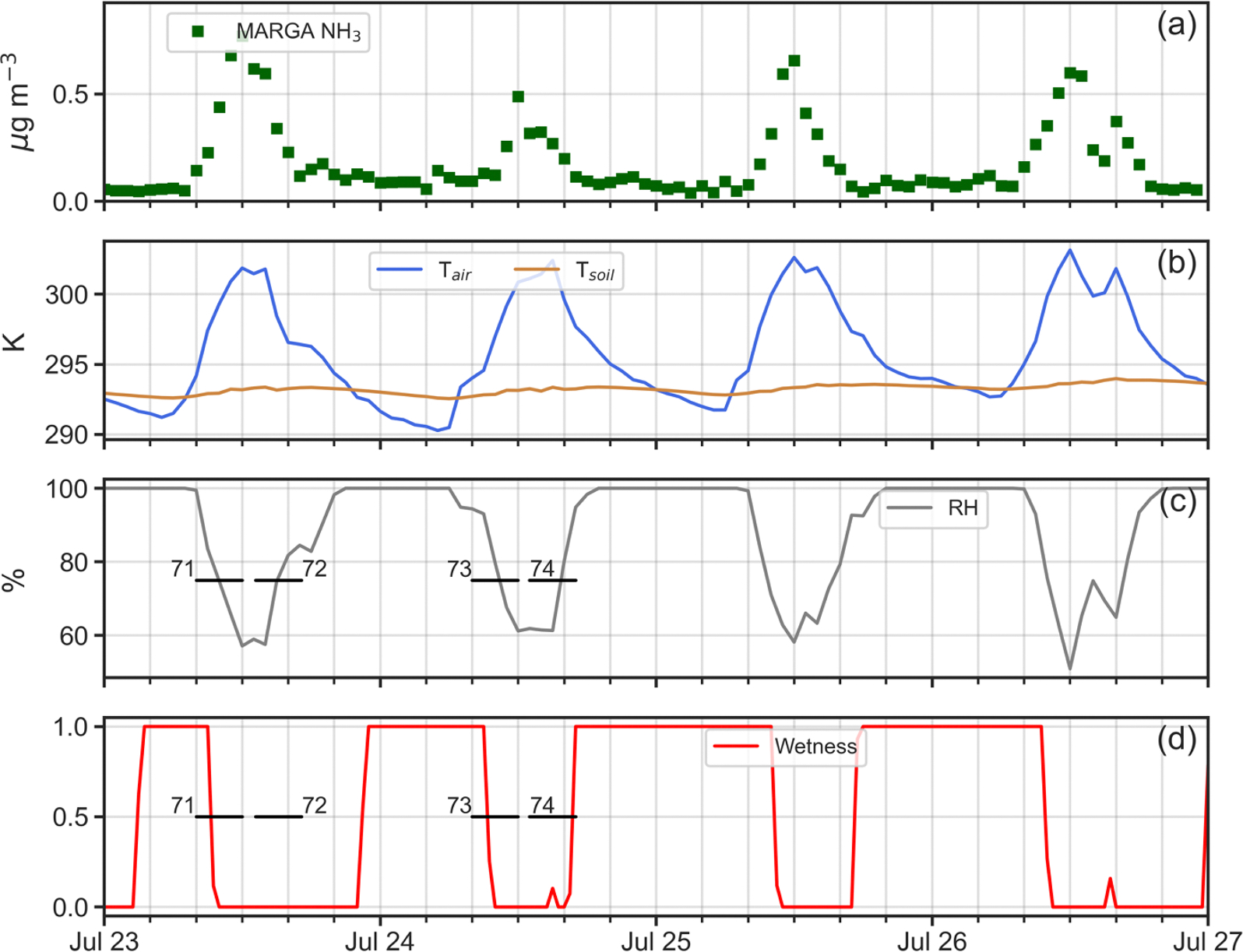
Time series plots of (a) adjusted MARGA ${NH}_{3}$ concentrations (μg m^−3^); (b) in-canopy (20 m AGL) air temperature (K) and 5 cm soil temperature (K); (c) in-canopy (20 m AGL) relative humidity (%); and, (c) canopy wetness index at 32 m AGL (0–1) for July 23–27, 2016. Temporal extent of denuder-based ${NH}_{3}$ profiles 71, 72, 73 and 74 shown as horizontal black lines.

**Fig. 12.**
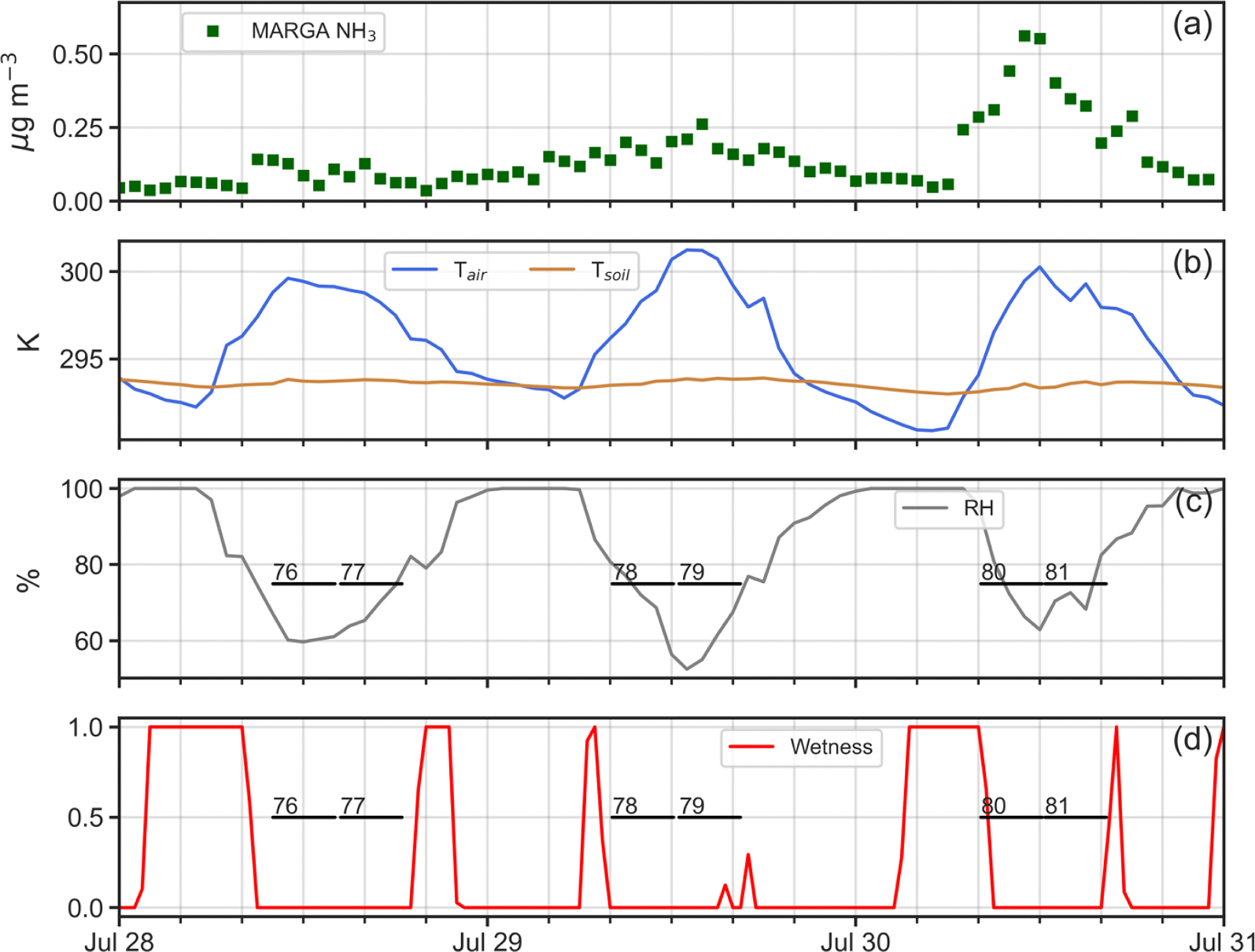
Time series plots of (a) adjusted MARGA ${NH}_{3}$ concentrations (μg m^−3^); (b) in-canopy (20 m AGL) air temperature (K) and 5-cm soil temperature (K); (c) in-canopy (20 m AGL) relative humidity (%); and, (c) canopy wetness index at 32 m AGL (0–1) for July 28–30, 2016. Temporal extent of denuder-based ${NH}_{3}$ profiles 76, 77, 78, 79, 80 and 81 shown as horizontal black lines.

In contrast, in Fig. 8 for Profiles 65, 71, 73 and 80, the model does a poor job of replicating the denuder concentrations, with generally higher observed values than simulated by the model through much of the canopy. As seen in Figs. 9–12, rapid increases in the above canopy ${NH}_{3}$ concentrations as measured by the MARGA instrument often coincide with rapid drops in relative humidity and canopy wetness, suggesting that ${NH}_{3}$ absorbed into dew overnight is released as the dew evaporates the following morning. Profiles 65, 71, 73 and 80 were all obtained during periods of rapid canopy drying, whereas Profiles 67, 68, 72, 74, 76, 77, 78 and 79 were obtained during periods with no evidence of dew evaporation. The process of ${NH}_{3}$ absorption/emission into/from dew is not currently included in the model, which apparently leads to poorer performance during periods of rapid canopy drying.

It has been recognized for some time that the occurrence of dew or guttation on vegetative canopies may have an impact on ${NH}_{3}$ bi-directional exchange, with the formation of dew resulting in ${NH}_{3}$ absorption, while subsequent dew evaporation results in re-emission (Denmead et al., 1976; Harper et al., 1983; van Hove et al., 1989; Bussink et al., 1996; Wyers and Erisman, 1998). More recently, with the common availability of higher temporal resolution measurements, a morning peak in ${NH}_{3}$ concentration has been observed in field measurements over a variety of vegetation types in many different locales (Walker et al., 2006; Wichink Kruit et al., 2007; Bash et al., 2010; Ellis et al., 2011; Wentworth et al., 2014; Kuang et al., 2020; He et al., 2020). Dew evaporation has been recognized in most of these studies as being a possible explanation for the morning maximum; however, other explanations have also been put forward. Other hypotheses for morning peaks in ${NH}_{3}$ concentration include: (i) emissions from plant stomata; (ii) emissions from the soil or ground litter as ambient temperature rises; (iii) downward mixing from a previous day’s residual layer as the PBL grows; or, (iv) in some locations, emissions from vehicles during morning rush hour. In our case, for the Coweeta SANDS dataset, although these other explanations cannot be ruled out completely, the good performance of the model during periods of a dry canopy and the poor performance during periods of canopy drying give strong credence to the hypothesis that ${NH}_{3}$ release from dew evaporation is the cause of strong morning concentration peaks.

A simple calculation can be performed to estimate the maximum morning ${NH}_{3}$ concentration that might result from instantaneous evaporation of dew that has come to equilibrium with an overnight ambient canopy air ${NH}_{3}$ concentration $\chi_{{NH}_{3}}^{amb}$. Assuming a canopy dew content $\theta_{\text{dew}}$, the maximum canopy air ${NH}_{3}$ concentration is given by

(16) $$\chi_{NH_{3}}^{max}=\chi_{NH_{3},T}^{eq}\theta_{\mathrm{dew}}$$

where, $\chi_{{NH}_{3},T}^{eq}$ is the equilibrium total ${NH}_{3}$ concentration in dew exposed to an overnight ambient ${NH}_{3}$ concentration $\chi_{{NH}_{3}}^{amb}$.

The canopy dew content is the product of an effective dew depth, $d_{\mathrm{eff}}$, on the leaves in the canopy and the leaf area density, LAD,

(17) $$\theta_{\mathrm{dew}}=d_{\mathrm{eff}}LAD,$$

while the effective dew depth is given by

(18) $$d_{eff}=W_{\mathrm{dew}}/\rho_{w},$$

where, $W_{\mathrm{dew}}$ is mean dew mass loading on leaves, and $\rho_{w}$ is water density.

The equilibrium absorption of gaseous of ${NH}_{3}$ into dew on leaf surfaces is governed by Henry’s Law

$${NH}_{3}\left(g\right)\overset{H_{NH}}{↔}{NH}_{3}\left(aq\right),$$

where, $H_{NH_{3}}$ is Henry’s Law constant for ${NH}_{3}$ in water. But, aqueous ${NH}_{3}$ can form ammonium ions in the dew via

$${NH}_{3}\left(aq\right)+H_{2}O\overset{K_{NH_{3}}}{↔}{NH}_{4}^{+}\left(aq\right)+{OH}^{-}\left(aq\right),$$

which leads to an effective Henry’s Law constant, $H_{NH_{3}}^{\mathrm{eff}}$, as a function of the ${NH}_{3}$ dissociation constant, $K_{{NH}_{3}}$, and the acidity of the dew

(19) $$H_{NH_{3}}^{\mathrm{eff}}=H_{NH_{3}}\left(1+\frac{K_{NH_{3}}\left[H^{+}\right]}{K_{w}}\right).$$

Using the ideal gas law and $H_{{NH}_{3}}^{\mathrm{eff}}$, the equilibrium total concentration in dew exposed to ambient ${NH}_{3}$ concentration $\chi_{{NH}_{3}}^{amb}$ is given by

(20) $$\chi_{NH_{3},T}^{eq}=\chi_{NH_{3}}^{amb}R_{g}T_{\mathrm{air}}H_{NH_{3}}^{eff}.$$

Then, combining Eqs. (16)–(20), the final result is

(21) $$\chi_{NH_{3}}^{max}=\chi_{NH_{3}}^{amb}R_{g}T_{\mathrm{air}}H_{NH_{3}}\left(1+\frac{K_{NH_{3}}\left[H^{+}\right]}{K_{w}}\right)W_{\mathrm{dew}}LAD/\rho_{w},$$

showing that the maximum canopy air ${NH}_{3}$ concentration that can be generated from dew evaporation is a function of the overnight ambient ${NH}_{3}$ concentration, the air temperature, the pH and mass loading of dew on the leaves, and the canopy leaf area density.

**Fig. 13.**
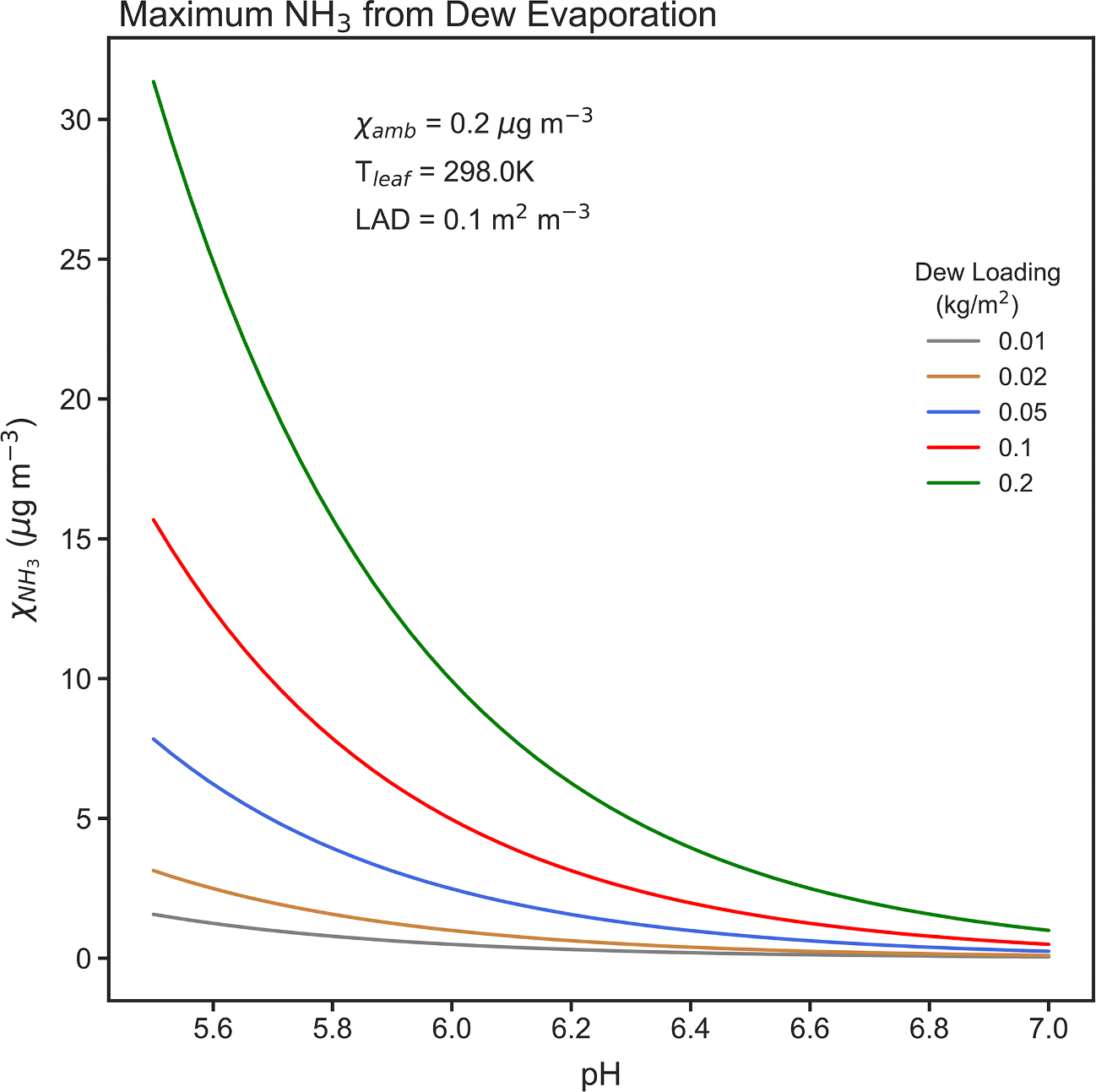
Maximum ${NH}_{3}$ concentrations (μg m^−3^) that can be obtained from complete evaporation of dew in a canopy as a function of pH and dew loading, given an overnight ambient concentration of 0.2 μg m^−3^, a leaf temperature of 298 K and a leaf area density of 0.1 m^2^ m^−3^.

A survey of the literature reveals relatively consistent dew loading ranges for a variety of vegetation types of 0.05–0.3 kg m^−2^ (Chameides, 1987), 0.05–0.4 kg m^−2^ (Wesely et al., 1990), < 0.15 kg m^−2^ (Wentworth et al., 2016), and 0.02–0.1 kg m^−2^ (Gerlein-Safdi et al., 2018). Leaf area density varies considerably between evergreen and broadleaf canopy types, but usually falls somewhere in the range 0.02–0.4 m^2^ m^−3^. Dew pH measurements typically lie within a broad range of 4.5–8.00 (Wesely et al., 1990; Lekouch et al., 2010; Wentworth et al., 2016), likely depending on the quantity and chemical nature of material previously dry deposited on the leaf, but a range of 5.5–7.0 gives a reasonable representation of potential ${NH}_{3}$ concentrations from dew evaporation. Fig. 13 provides estimates of maximum ${NH}_{3}$ concentrations (μg m^−3^) that can be obtained from complete evaporation of dew in a canopy as a function of pH and dew loading. These equilibrium estimates assume an overnight ambient concentration of 0.2 μg m^−3^, a leaf temperature of 298 K and a leaf area density of 0.1 m^2^ m^−3^. From a different perspective, the maximum dew emission potential, $\Gamma_{dew}^{max}$, which is a function of $\chi_{NH_{3}}^{amb}$ and $T_{\mathrm{air}}$, ranges from 14.6 at an ambient ${NH}_{3}$ concentration of 0.1 μg m^−3^ and an air temperature of 25 °C, to 73.2 at 0.5 μg m^−3^ and 25 °C. From these back-of-the-envelope estimates, it seems clear that rapid dew evaporation in the early morning hours, as seems to be happening at Coweeta, could indeed generate bursts of increased ${NH}_{3}$ concentrations within and above the forest canopy, especially if dew pH < 6.5. On the other hand, if ${NH}_{3}$ absorbed into the overnight dew forms low- or semi-volatile salts, then evaporation of the dew may not result in complete liberation of the captured ${NH}_{3}$, leaving ammonium salts on the dry leaf surface. Thus, the estimates of potential gas-phase ${NH}_{3}$ from dew evaporation in Fig. 13 are upper bounds, dependent on the relative concentrations of anions versus cations resident in the dew (Wentworth et al., 2016; Takenaka et al., 2009).

In order to determine model sensitivities, simulations were performed for all time periods in which key model parameters were varied across expected ranges of values. Four model parameters were found to have the most impact on the magnitude and shape of in-canopy ${NH}_{3}$ profiles. These included the soil/litter emission potential, $\Gamma_{g}$, the stomatal emission potential, $\Gamma_{s}$, the litter resistance, $r_{litter}$ and an eddy diffusivity scaling factor, $K_{\mathrm{sens}}$. For each time period, an optimal set of parameters (Table 1) were selected so that the simulated mean ${NH}_{3}$ concentration profile best represented a majority of the observed profiles measured by in-canopy denuders. For the soil/litter emission potential, the field measured value of 200 provided the best results for the July time periods; however, for April, any non-zero value introduced a near-ground increase in ${NH}_{3}$ concentrations that was not observed in the denuder measurements. Overall, the values of the stomatal emission potential had less of an impact on in-canopy profiles than the other parameters, but a value of 20 (within the range of measured values) produced the best results for all time periods.

**Fig. 14.**
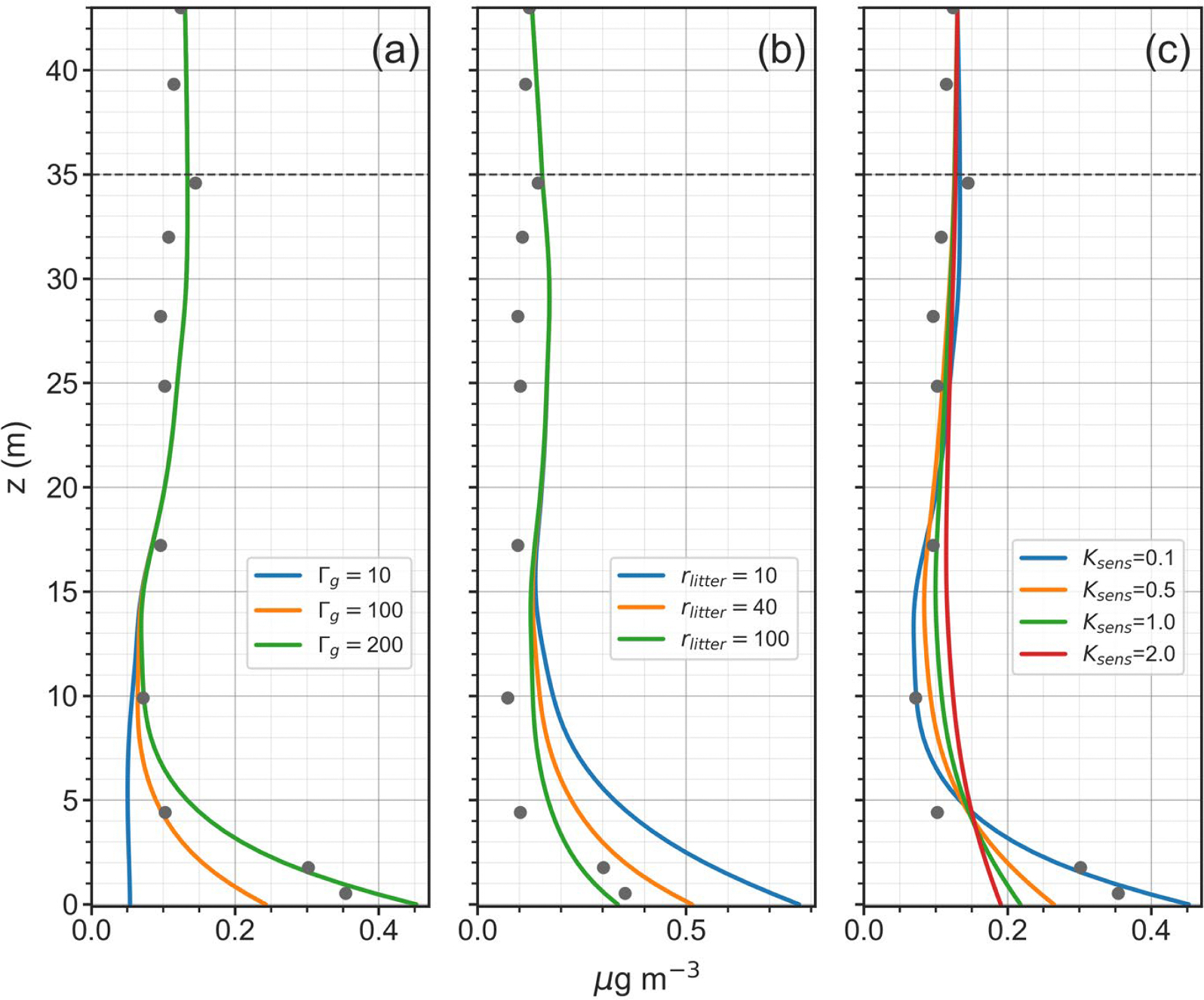
Sensitivities of mean modeled ${NH}_{3}$ concentration profiles (μg m^−3^) to values of (a) the input soil/litter emissions potential, $\Gamma_{g}$; (b) the litter resistance $r_{litter}$ (s cm^−1^); and, (c) the eddy diffusivity sensitivity factor $K_{\mathrm{sens}}$. Model runs and observations are for Profile #77, 2:00–6:00PM on July 28, 2016.

Results for model sensitivities to $\Gamma_{g},\;r_{\mathrm{litter}}$, and $K_{\mathrm{sens}}$ are shown in Fig. 14. As would be expected, the soil/litter emissions potential and the litter resistance have a large impact on the ${NH}_{3}$ concentration profile in the lowest 5 m above the forest floor. Increasing $\Gamma_{g}$ or decreasing $r_{\mathrm{litter}}$ serves to increase the concentration gradient near the surface. As $\Gamma_{g}$ approaches zero, the vertical gradient also approaches zero. The litter resistance, however, is more uncertain, but still significantly affects the near-ground concentration gradient. There are few studies that have tried to directly measure litter resistance and its value would be expected to vary greatly according to a number of factors, including season, forest density, tree and understory species and decay rate. The last important input parameter is the in-canopy eddy diffusivity profile. In the sensitivity simulations, the $K_{v}^{\mathrm{cnpy}}$ profile generated according to Eqs. (1)–(9) in Supplement I was altered by applying a sensitivity factor to the entire profile. As seen in Fig. 14c, realistic gradients near the forest floor were only obtained when the eddy diffusivity profile was reduced by a factor of ten or more. This result was consistent across all simulation periods and suggests that standard within-canopy formulations of eddy diffusivities need to be substantially altered or replaced with alternative representations to properly represent within-canopy turbulent transport, especially near the forest floor.

## 4. Summary and findings

Chemical concentration and flux measurements of ${NH}_{3}$ made in 2016 at the western North Carolina Coweeta Hydrologic Laboratory during SANDS have been analyzed using a multi-layer, one-dimensional column model (ACCESS-NH3) with detailed canopy physics and bidirectional exchange of ${NH}_{3}$. Simulations for two time periods in 2016, April 26–30 and July 19–30, are presented with the goal of assessing the model’s ability to reasonably represent the measured in-canopy profiles of ${NH}_{3}$ and probe the processes that control the bidirectional exchange of ${NH}_{3}$ with the canopy and forest floor.

Generally, the multi-layer canopy model is found to produce reasonable ${NH}_{3}$ concentration profiles, with results falling into two categories. Given appropriate inputs and during dry canopy conditions, ACCESS-NH3 simulations are found to well reproduce measured ${NH}_{3}$ in-canopy profiles for both the April and July periods. Results from the model, and the shape of in-canopy ${NH}_{3}$ profiles in particular, are sensitive to vertical turbulent mixing (i.e., the magnitude of eddy diffusivity vertical profiles, $K_{v}^{\mathrm{cnpy}}$), the values of the input soil/litter emission potential, $\Gamma_{g}$, and the assumed litter resistance, $r_{litter}$. Overall, ${NH}_{3}$ fluxes simulated above the canopy are very small (−25 to −5 ng m^−2^ s^−1^ in April and < 1 ng m^−2^ s^−1^ in July) with primarily deposition to the canopy during the April time period, but with mixed deposition/emission during July. Within the canopy, the model suggests that deposition or emission of ${NH}_{3}$ varies with location within the canopy. In April, when deposition predominates, the highest deposition rates occur in the upper canopy with rates diminishing towards the forest floor. In July, the situation is more complex, often with deposition in the understory where the highest ${NH}_{3}$ concentrations occur, but with either deposition or emission occurring in the mid- to upper-canopy depending on the difference between the air concentration and the effective canopy compensation point.

On the other hand, the model does a worse job of replicating in-canopy ${NH}_{3}$ profiles during times when the canopy is rapidly drying. Data from the MARGA above the canopy typically exhibit rapid bursts of ${NH}_{3}$ as measured canopy wetness decreases quickly during these periods. Although the data available during the SANDS experiment is not sufficient to rule out conclusively other potential hypotheses for the observed ${NH}_{3}$ concentration bursts, a simple equilibrium calculation suggests that rapid drying of canopy dew formed overnight has the potential to release sufficient ${NH}_{3}$ to account for the observed morning concentration bursts. Given that the model reasonably reproduces concentration profiles during dry periods but fails during periods of dew evaporation (generally underpredicting ${NH}_{3}$ concentrations since the model does not account for overnight dew absorption or morning evaporative emission), the results are suggestive that dew is playing a major role in ${NH}_{3}$ concentration changes observed during July at Coweeta.

Results from numerous sensitivity runs of the model (not all reported here as significant) have provided several insights into the physical and environmental parameters that impact ${NH}_{3}$ surface-atmosphere exchange. Differences between the in-canopy profile shapes between April and July seem to be driven by the lack of significant ${NH}_{3}$ emission from the soil/litter in April, while a strong surface source is required to adequately reproduce the concentration gradient observed near the forest floor in July. An important, but highly uncertain, component of the model is the magnitude of the litter resistance, $r_{litter}$. As noted in Fig. 14(b), the value of the resistance serves to modulate the amount of ${NH}_{3}$ that is released from the soil/litter, directly affecting the severity of the near-surface concentration gradient. Little information is available about the magnitude of $r_{litter}$ or how to calculate it, suggesting further measurements and theoretical development are necessary for this critical parameter. Additional thought and possibly new measurements are also needed to better understand scalar transport in dense forest canopies, especially in the understory. Although it is well known that gradient transport theory is not truly applicable for modelling turbulence in this environment, we have demonstrated that it can produce realistic results under some conditions. Given that forest floors are often involved in the emission or uptake of a variety of climatic or air quality related trace species (e.g., carbon dioxide, methane, nitric oxide, ${NH}_{3}$, etc.), an improved theoretical understanding of near-surface exchange and transport is needed.

The findings of this analysis, in conjunction with other studies (Walker et al., 2006; Wichink Kruit et al., 2007; Bash et al., 2010; Ellis et al., 2011; Wentworth et al., 2014; Kuang et al., 2020; He et al., 2020), clearly indicate a need to better understand the dynamics of ${NH}_{3}$ interaction with dew and guttation processes in vegetative canopies. Additional measurements are needed to pinpoint the processes and environmental controls that affect ${NH}_{3}$ absorption and release from dew and to evaluate the importance of this process for modelling ${NH}_{3}$ deposition/emission on the regional scale. In particular, measurements of in-canopy ${NH}_{3}$ concentrations are needed during periods of dew formation and canopy drying with enough time resolution to evaluate the hypothesis of ${NH}_{3}$ uptake and release by dew. Measurements of dew chemistry coinciding with air concentration observations would also be helpful.

A question that arises from our findings is whether the variation of ${NH}_{3}$ deposition or emission with location in the canopy is important from an ecological perspective. One example of the possible ecological importance might be that vegetation overstory and understory species may have different tolerances for deposition, based on their ability to assimilate ${NH}_{3}$ into the leaf apoplast. Some understory species, such as rhododendrons, tend to have lower than average emission potentials as compared to typical overstory species (Walker et al., 2023). If vertical variation of emissions/deposition does turn out to be ecologically important for some species or particular ecosystems, then another question is raised on how best to represent in-canopy dynamics in regional-scale air quality models.

The ”big-leaf” approach to modelling surface-atmosphere exchanges represents vegetative canopies as single layers with no vertical structure or variation. A more discriminating version divides the modelled canopy into sunlit and shaded fractions allowing for different environmental conditions within each partition. This simple approach served well when atmospheric models employed horizontal grid resolutions of 20–40 km or even larger. Today, as state-of-the-science models use much finer horizontal resolutions, some have advocated moving away from the bigleaf approach towards multi-layer treatments of vegetative canopies, especially for forests (Bonan et al., 2024, 2021, 2018). Results from this study suggest that traditional big-leaf approaches of modelling ${NH}_{3}$ bi-directional exchange cannot account for in-canopy variation such as that modelled here, and so multi-layer approaches may need to be developed for more nuanced estimates of ${NH}_{3}$ deposition/emission in large-scale atmospheric models.

## Supplementary Material

Supplement1

## Figures and Tables

**Table 1:** Domain and Simulation Parameters for the 2016 Coweeta Hydrologic Laboratory measurement site.

Simulation dates	Soil/litter emission potential, $\Gamma_{g}$	Stomatal emission potential, $\Gamma_{s}$	Litter resistance, $r_{\mathrm{litter}}$ (s/cm)

April 26–30, 2016	0	20	2
July 19–21, 2016	200	20	40
July 23–26, 2016	200	20	40
July 28–30, 2016	200	20	40

H= domain top (m);= 37 m for April period;= 43 m for July periods.

Δz= grid resolution (m)= 0.5 m.

LAI= leaf area index= 3.3 for April and= 4.6 for July.

$h_{c}$= canopy height (m)= 35 m.

## Data Availability

Model code and results are available from R. Saylor (rick.saylor@noaa.gov) upon request. SANDS data are available from J. Walker (John.Walker3@usda.gov) upon request.
