# Supplementary material for: Dynamic ammonia exchange within a mixed deciduous forest canopy in the Southern Appalachians
Source: Ecol Modell. Author manuscript; Available in PMC 2026 Feb 1. (PMC12180929; doi:10.1016/j.ecolmodel.2024.111007)
Supplement: Supplement1 [file NIHMS2052333-supplement-Supplement1.zip › 1-s2.0-S0304380024003958-mmc2.pdf]

Supplement II to

Dynamic Ammonia Exchange within a Mixed Deciduous Forest Canopy in the Southern Appalachians

Rick D. Saylor[1], John T. Walker[2#], Zhiyong Wu[2], Xi Chen[2], Donna B. Schwede[3*], A. Christopher Oishi[4], and Nebila Lichiheb[1,5]

[1]National Oceanic and Atmospheric Administration, Air Resources Laboratory, Oak Ridge, TN 37830
[2]U. S. Environmental Protection Agency, National Risk Management Research Laboratory, Research Triangle Park, NC 27711
[3]U. S. Environmental Protection Agency, National Exposure Research Laboratory, Research Triangle Park, NC 27711
[4]U. S. Forest Service, Southern Research Station, Otto, NC 28763
[5]Oak Ridge Associated Universities, Oak Ridge, TN 37830

Current affiliations:
[#]USDA Forest Service, Southern Research Station, Center for Forest Watershed Research, Otto, NC 28763
*Retired

Figures S-1 through S-13

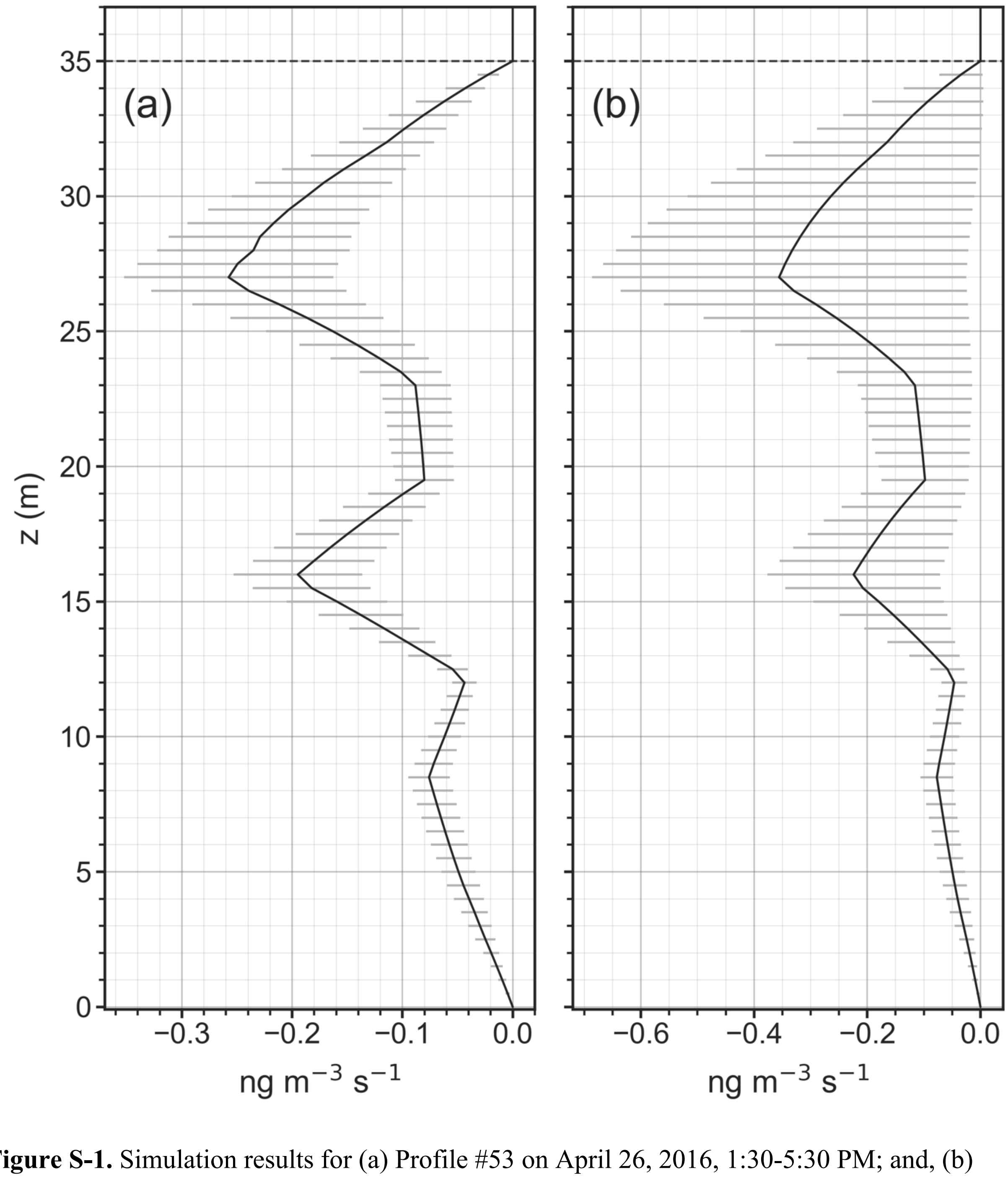

Figure S-1. Simulation results for (a) Profile #53 on April 26, 2016, 1:30-5:30 PM; and, (b) Profile #54 on April 27, 2016, 11:00 AM-3:00 PM. In each panel, the mean volumetric NH_3 source/sink (ng m^{-3} s^{-1}) profile (solid black line) is shown with ± 1 standard deviation (horizontal gray lines) over the measurement period. Horizontal dashed gray line denotes the canopy top.

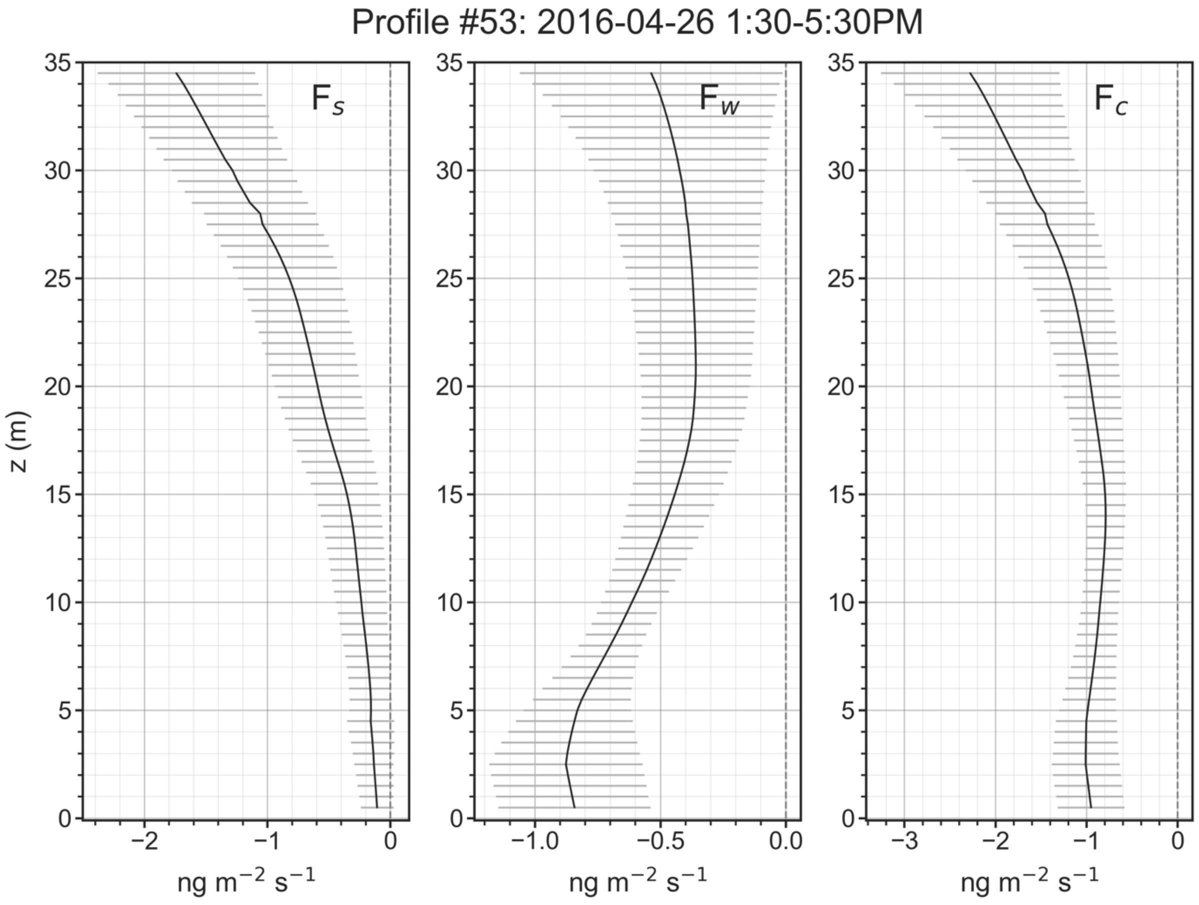

Figure S-2. Canopy profiles (solid black lines) for the stomatal flux (F_s), cuticular flux (F_w) and total canopy flux (F_c) (ng m^{-2} s^{-1}) for Profile #53 on April 26, 2016, 1:30-5:30 PM; ± 1 standard deviation shown as horizontal gray lines.

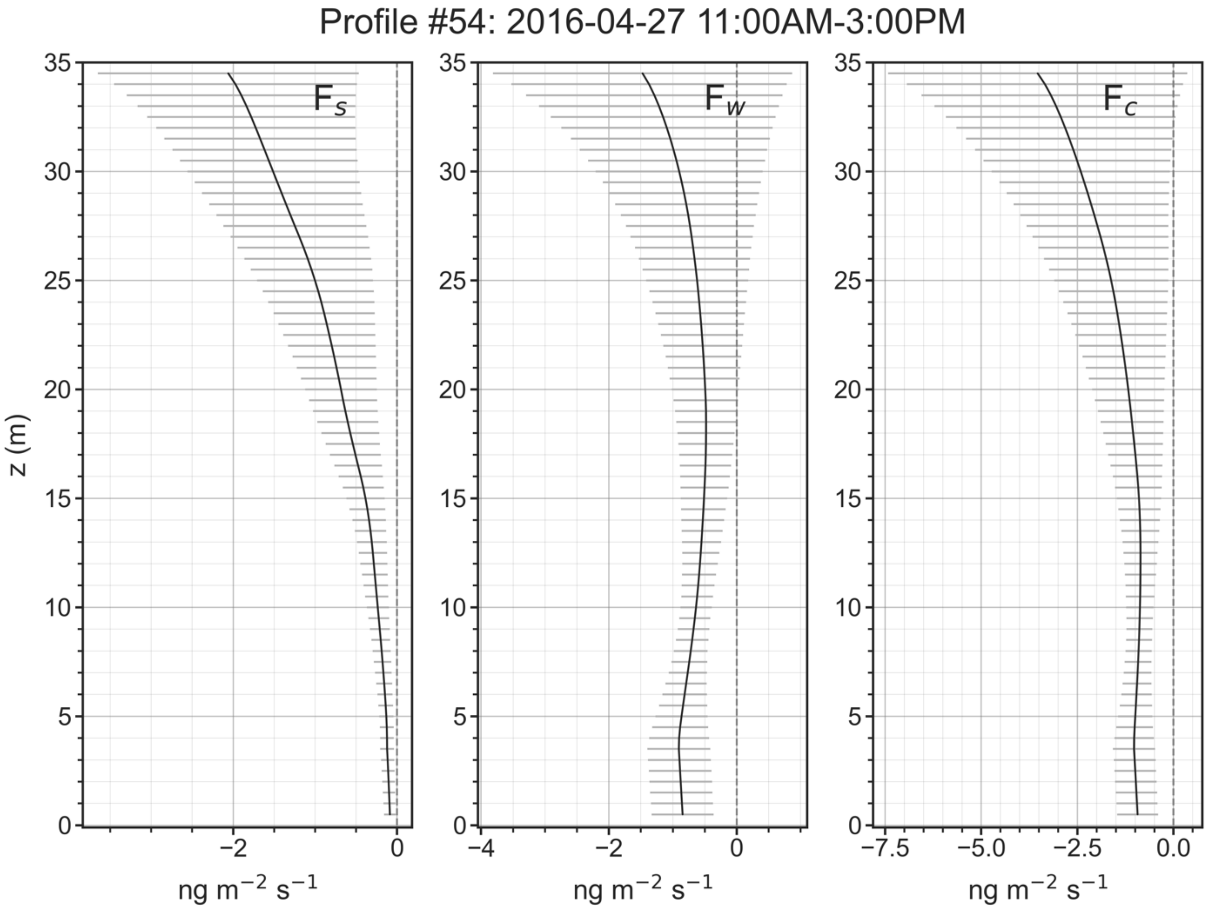

Figure S-3. Canopy profiles (solid black lines) for the stomatal flux (F_s), cuticular flux (F_w) and total canopy flux (F_c) (ng m^{-2} s^{-1}) for Profile #54 on April 27, 2016, 11:00 AM – 3:00 PM; ± 1 standard deviations shown as horizontal gray lines.

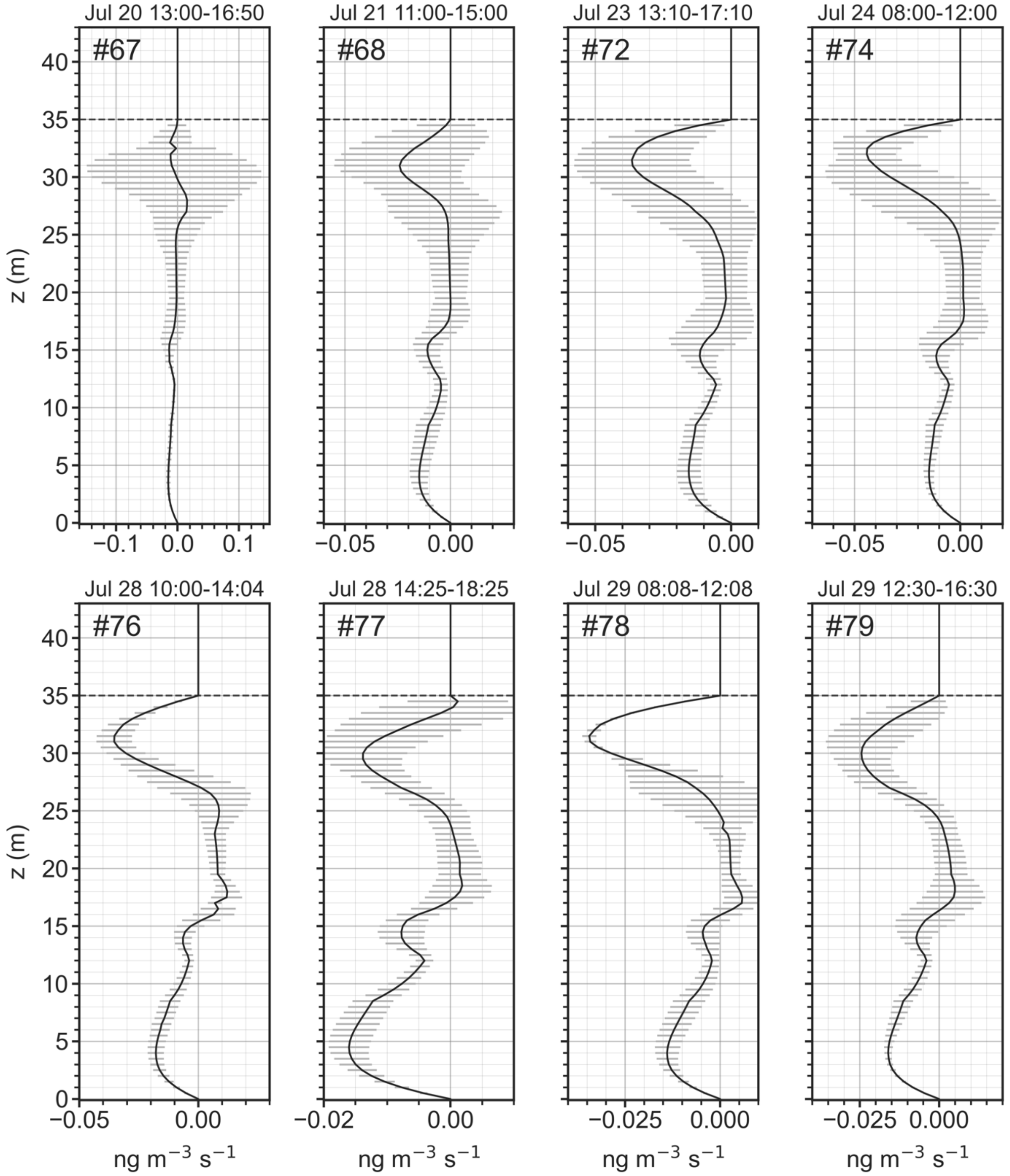

Figure S-4. Simulation results for profiles 67, 68, 72, 74, 76, 77, 78 and 79. In each panel, the mean volumetric NH_3 source/sink (ng m^{-3} s^{-1}) profile (solid black line) is shown with ± 1

standard deviation (horizontal gray lines) over the measurement period. Horizontal dashed gray line denotes the canopy top.

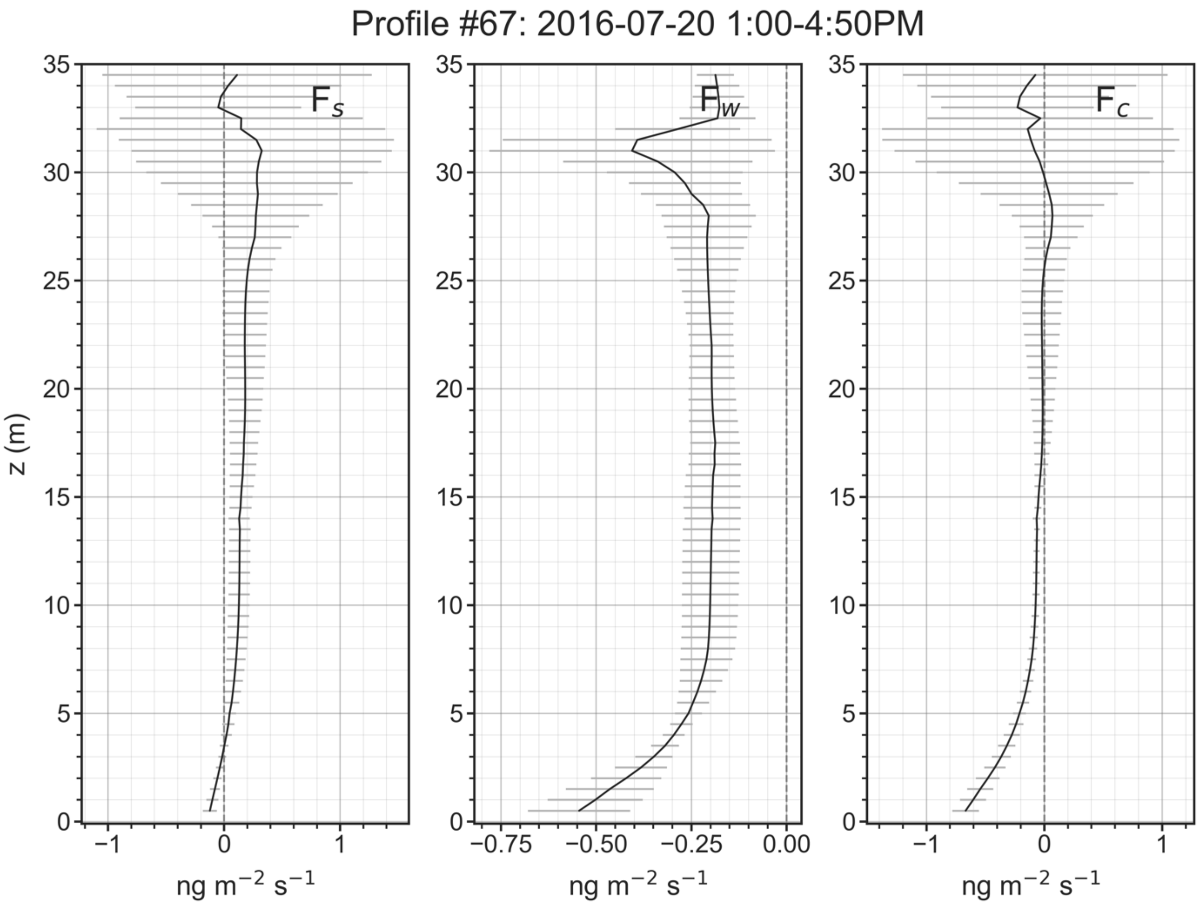

Figure S-5. Canopy profiles (solid black lines) for the stomatal flux (F_s), cuticular flux (F_w) and total canopy flux (F_c) (ng m^{-2} s^{-1}) for Profile #67 on July 20, 2016, 1:00–4:50 PM; ± 1 standard deviations shown as horizontal gray lines.

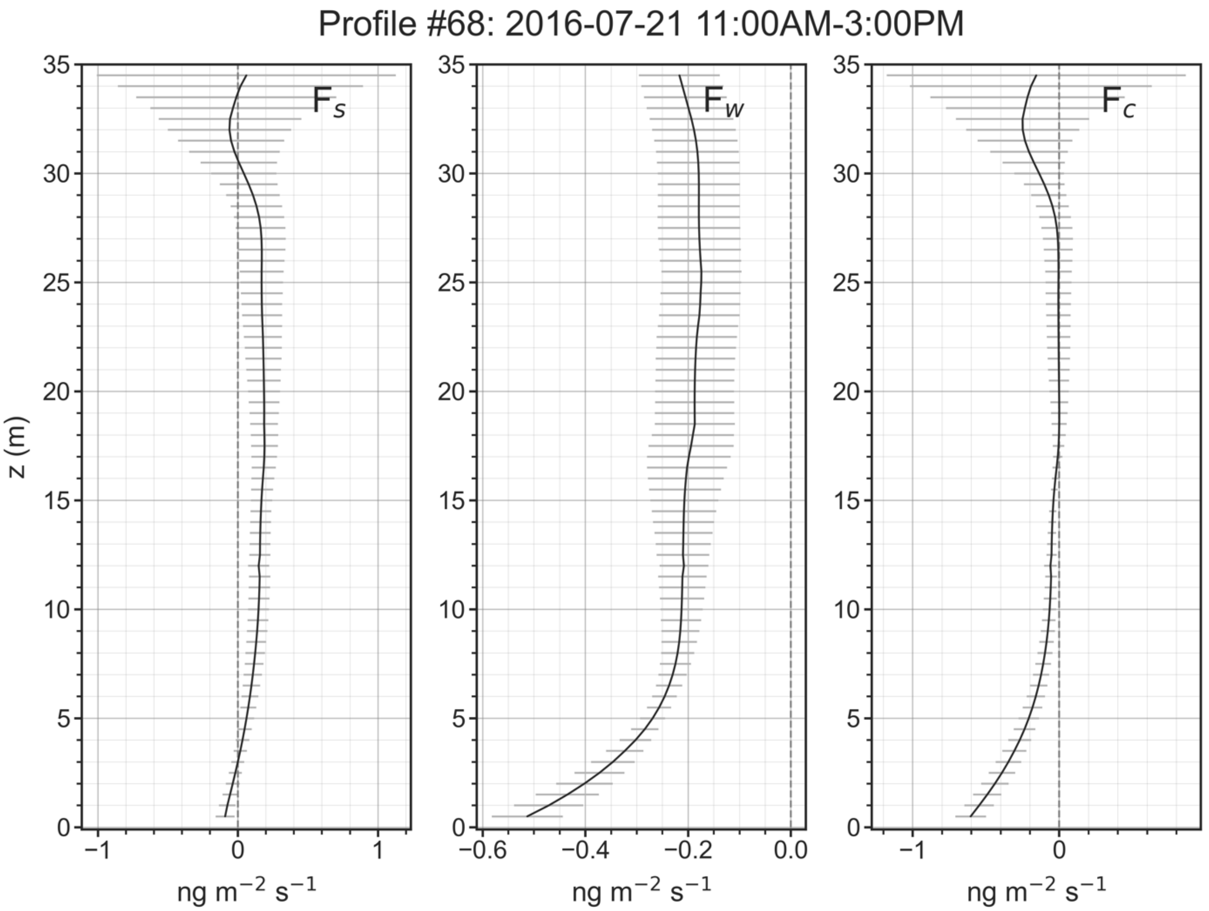

Figure S-6. Canopy profiles (solid black lines) for the stomatal flux (F_s), cuticular flux (F_w) and total canopy flux (F_c) (ng m^{-2} s^{-1}) for Profile #68 on July 21, 2016, 11:00 AM – 3:00 PM; ± 1 standard deviations shown as horizontal gray lines.

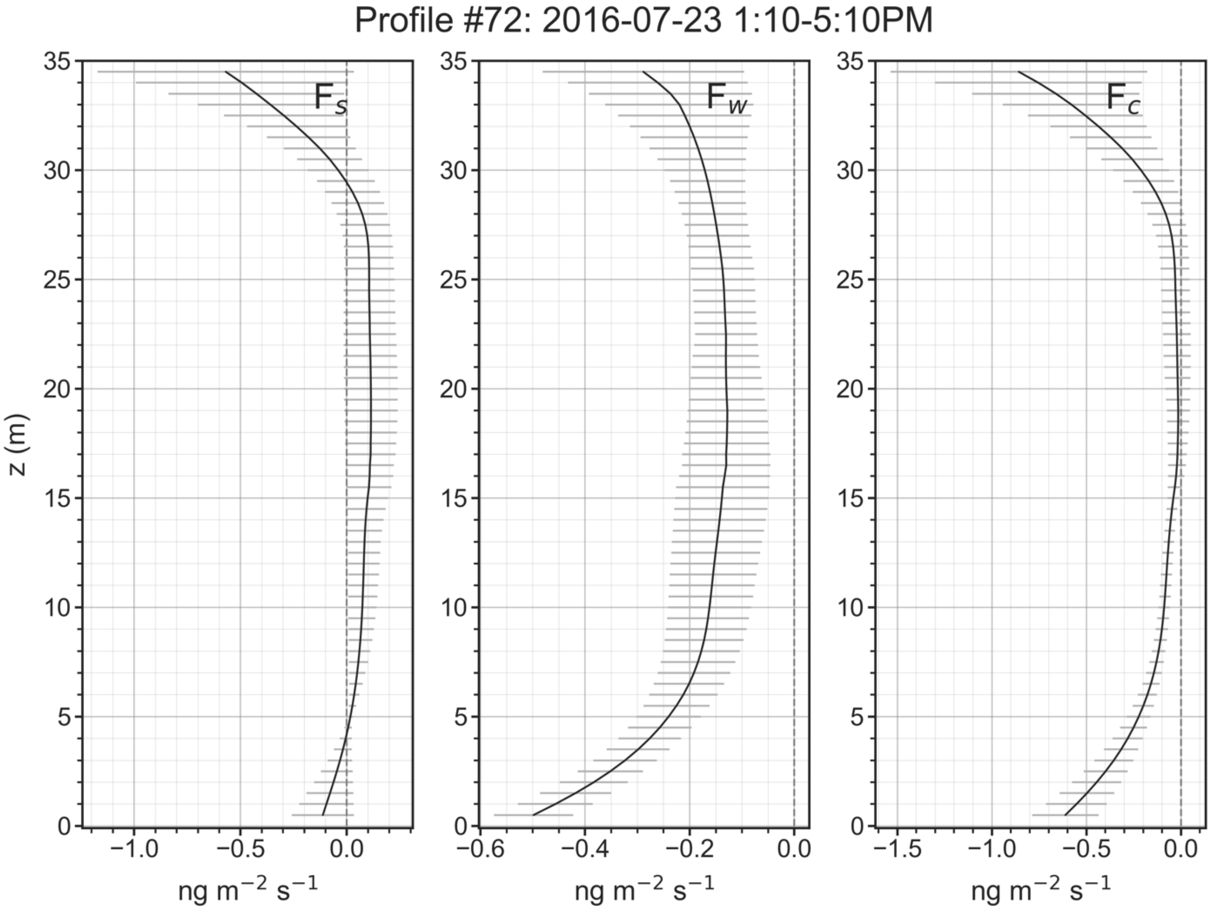

Figure S-7. Canopy profiles (solid black lines) for the stomatal flux (F_s), cuticular flux (F_w) and total canopy flux (F_c) (ng m^{-2} s^{-1}) for Profile #72 on July 23, 2016, 1:10 – 5:10 PM; ± 1 standard deviation shown as horizontal gray lines.

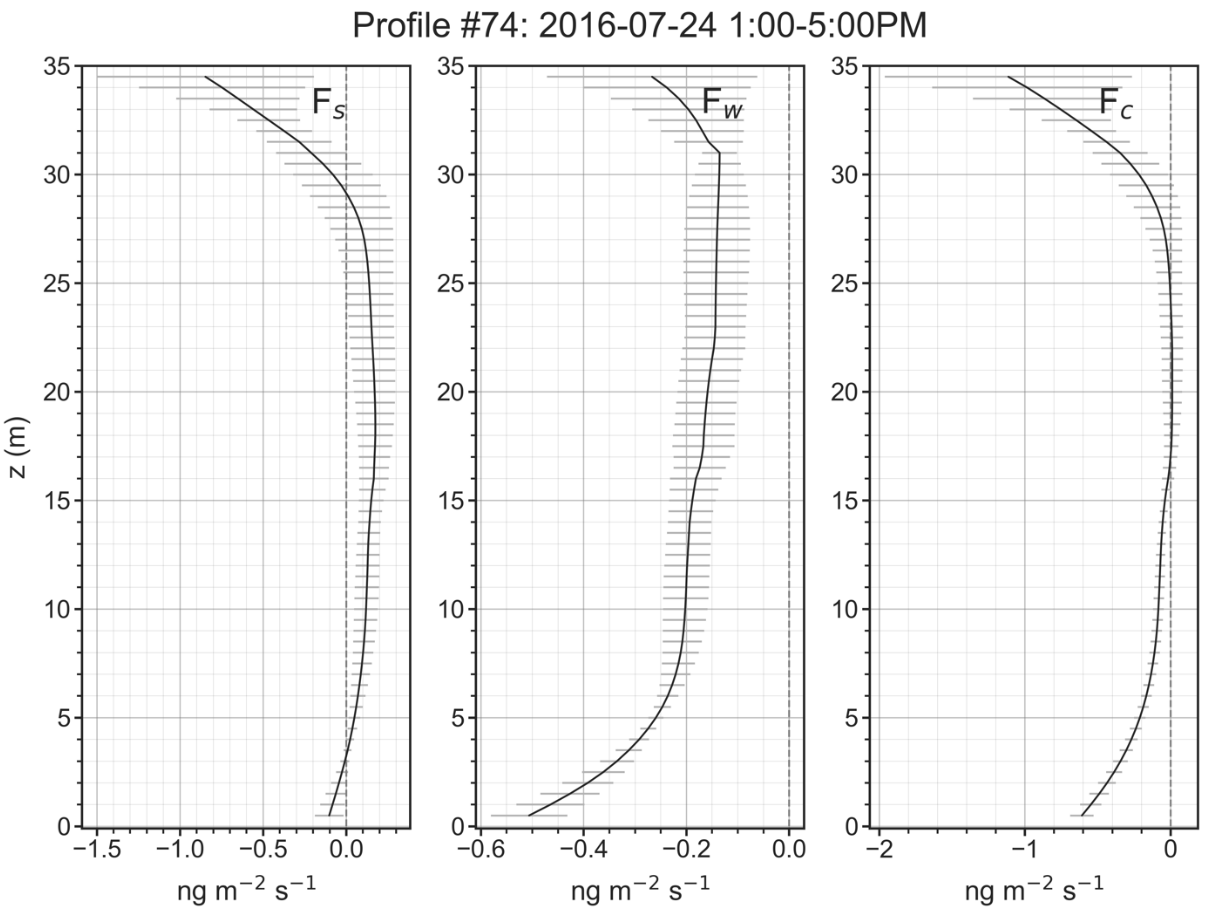

Figure S-8. Canopy profiles (solid black lines) for the stomatal flux (F_s), cuticular flux (F_w) and total canopy flux (F_c) (ng m^{-2} s^{-1}) for Profile #74 on July 24, 2016, 1:00 – 5:00 PM; ± 1 standard deviation shown as horizontal gray lines.

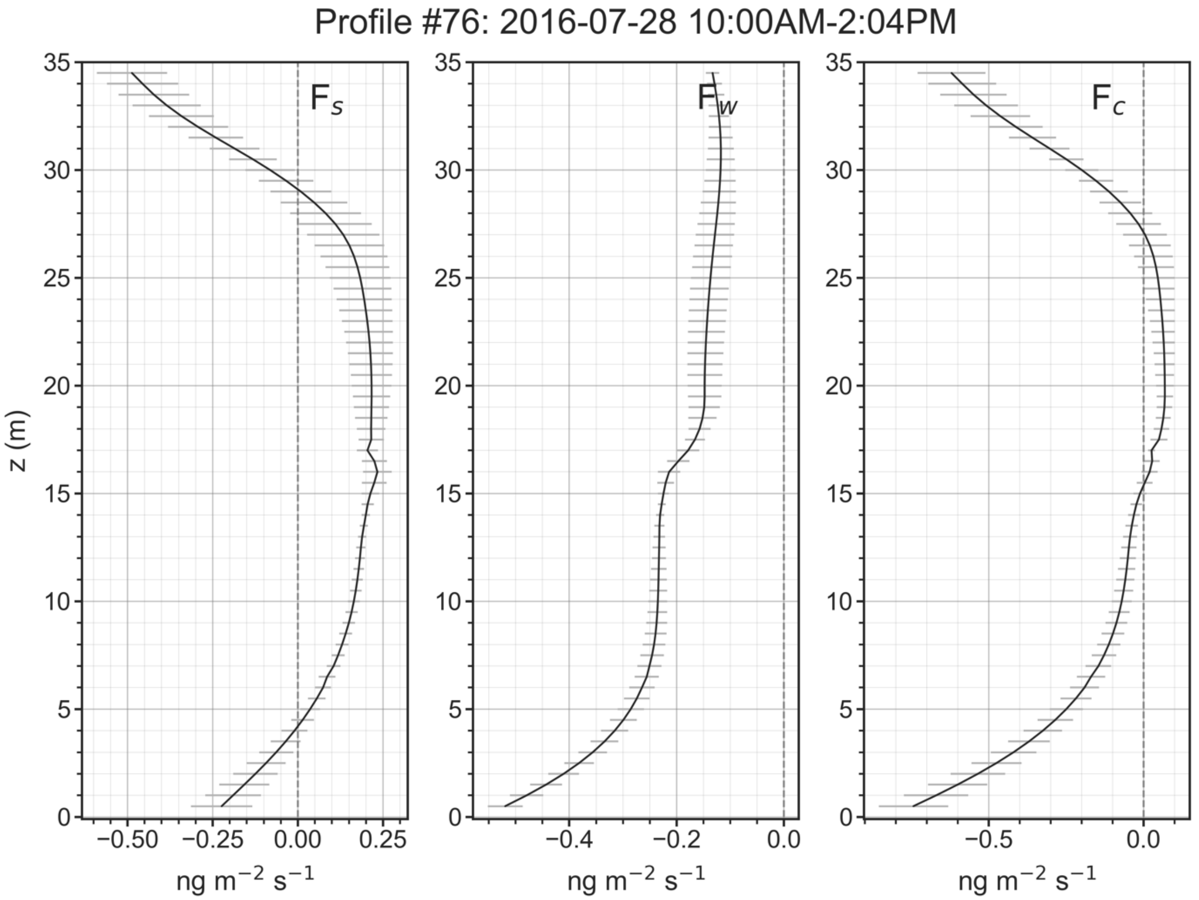

Figure S-9. Canopy profiles (solid black lines) for the stomatal flux (F_s), cuticular flux (F_w) and total canopy flux (F_c) (ng m^{-2} s^{-1}) for Profile #76 on July 28, 2016, 10:00 AM – 2:04 PM; ± 1 standard deviations shown as horizontal gray lines.

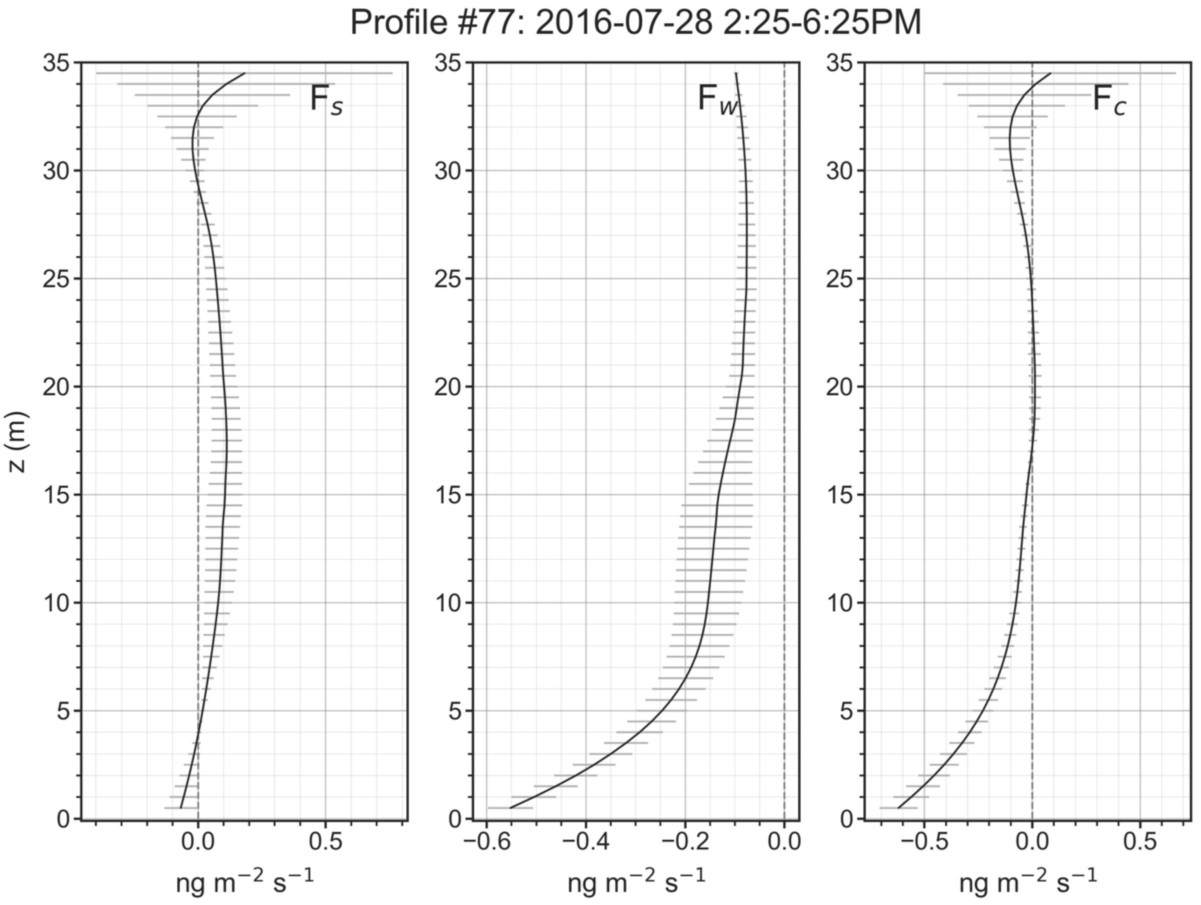

Figure S-10. Canopy profiles (solid black lines) for the stomatal flux (F_s), cuticular flux (F_w) and total canopy flux (F_c) (ng m^{-2} s^{-1}) for Profile #77 on July 28, 2016, 2:25–6:25 PM; ± 1 standard deviation shown as horizontal gray lines.

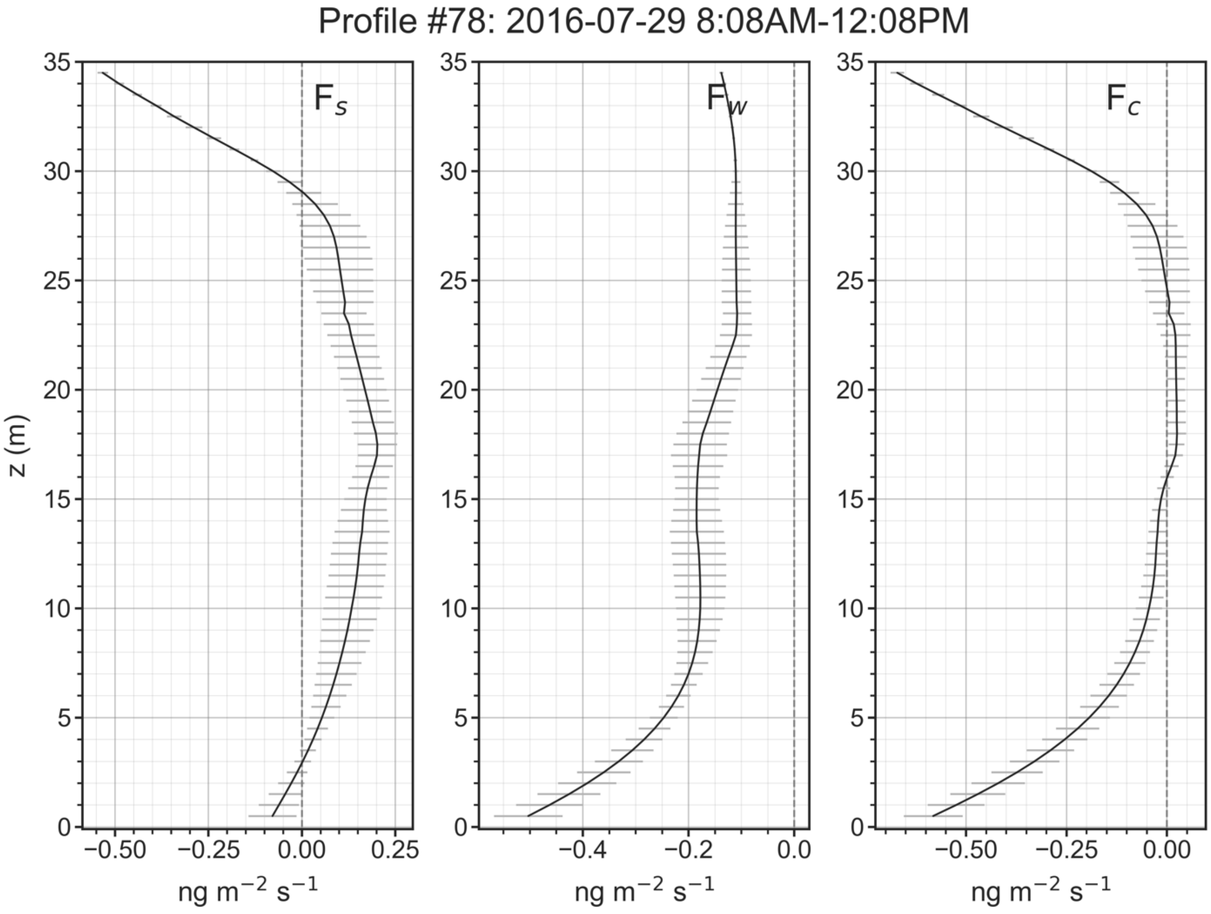

Figure S-11. Canopy profiles (solid black lines) for the stomatal flux (F_s), cuticular flux (F_w) and total canopy flux (F_c) (ng m^{-2} s^{-1}) for Profile #78 on July 29, 2016, 8:08 AM–12:08 PM; ± 1 standard deviation shown as horizontal gray lines.

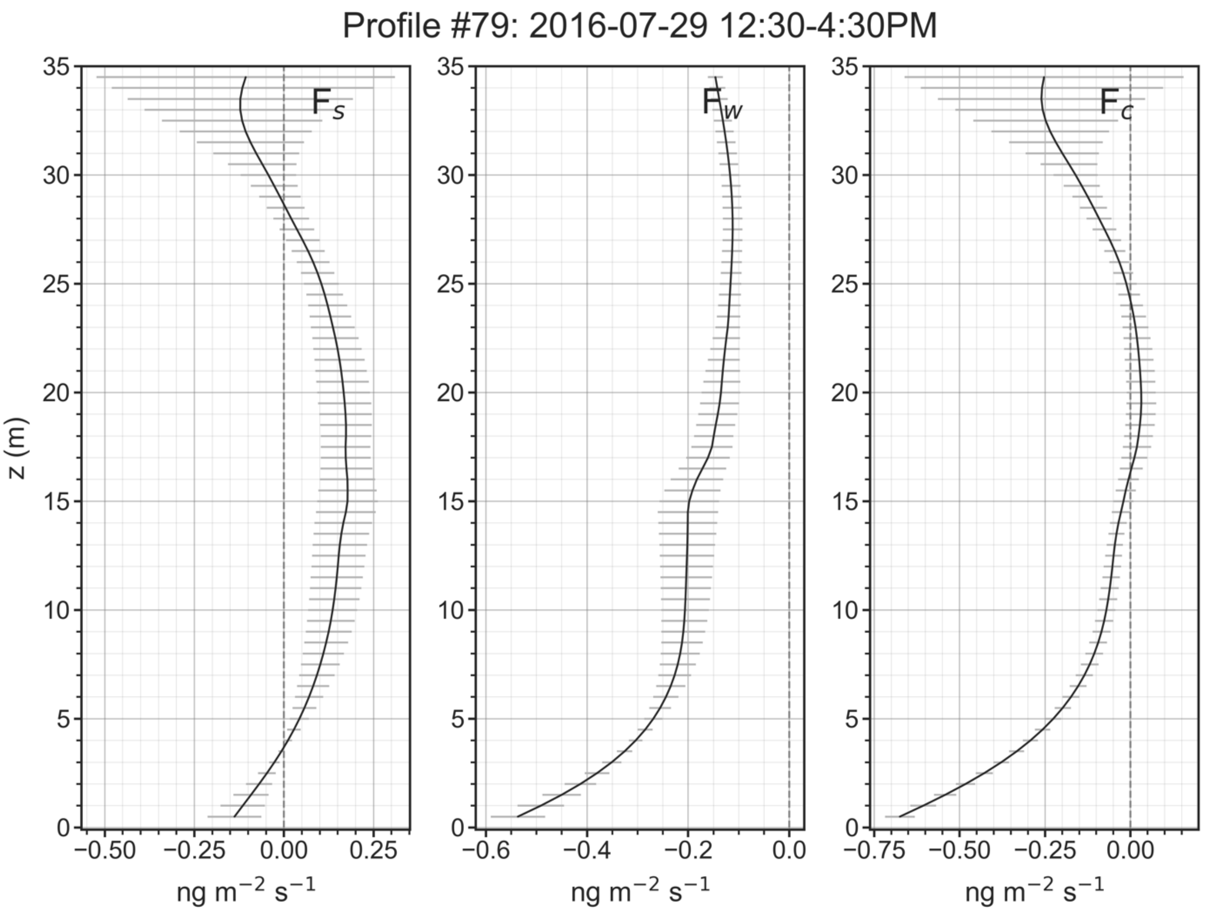

Figure S-12. Canopy profiles (solid black lines) for the stomatal flux (F_s), cuticular flux (F_w) and total canopy flux (F_c) (ng m^{-2} s^{-1}) for Profile #79 on July 29, 2016, 12:30–4:30 PM; ± 1 standard deviation shown as horizontal gray lines.

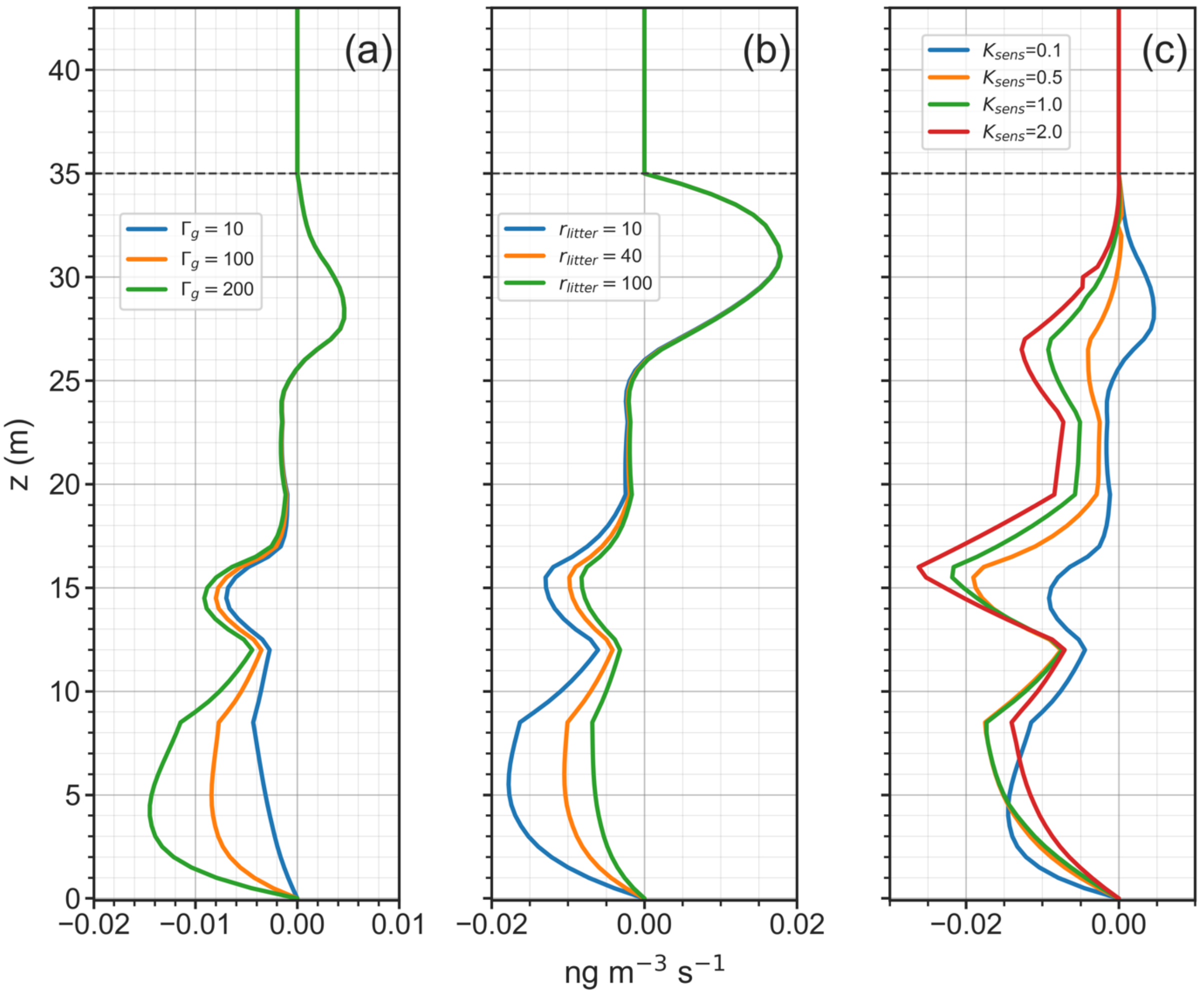

Figure S-13. Sensitivities of mean modeled NH_3 volumetric source/sink profiles (ng m^{-3} s^{-1}) to values of (a) the input soil/litter emissions potential; (b) the litter resistance (s cm^{-1}); and, the eddy diffusivity sensitivity factor. Model runs are for Profile #77, 2:00-6:00 PM on July 28, 2016.
