# Supplementary material for: Dynamic ammonia exchange within a mixed deciduous forest canopy in the Southern Appalachians
Source: Ecol Modell. Author manuscript; Available in PMC 2026 Feb 1. (PMC12180929; doi:10.1016/j.ecolmodel.2024.111007)
Supplement: Supplement1 [file NIHMS2052333-supplement-Supplement1.zip › 1-s2.0-S0304380024003958-mmc1.pdf]

Supplement I to

Dynamic Ammonia Exchange within a Mixed Deciduous Forest Canopy in the Southern Appalachians

Rick D. Saylor[1], John T. Walker[2#], Zhiyong Wu[2], Xi Chen[2], Donna B. Schwede[3*], and A. Christopher Oishi[4], Nebila Lichiheb[1,5]

[1]National Oceanic and Atmospheric Administration, Air Resources Laboratory, Oak Ridge, TN 37830
[2]U. S. Environmental Protection Agency, National Risk Management Research Laboratory, Research Triangle Park, NC 27711
[3]U. S. Environmental Protection Agency, National Exposure Research Laboratory, Research Triangle Park, NC 27711
[4]U. S. Forest Service, Southern Research Station, Otto, NC 28763
[5]Oak Ridge Associated Universities, Oak Ridge, TN 37830

Current affiliations:
[#]USDA Forest Service, Southern Research Station, Center for Forest Watershed Research, Otto, NC 28763
*Retired

Details of the Canopy Physics, Vertical Mixing, and Surface Exchange of ACCESS-NH3

1 Introduction

The Atmospheric Chemistry and Canopy Exchange Simulation System (ACCESS) is a general modeling system that can be used to simulate one-dimensional atmospheric chemistry, vertical turbulent transport and biosphere-atmosphere exchange processes from the earth's surface to the top of the planetary boundary layer (PBL), with multilayer representation of vegetative canopy physics and atmosphere-canopy interactions. The initial version of ACCESS (ACCESS v1) is described in Saylor (2013), while the second version of the modeling system is described in an unpublished manuscript available from the author. This document describes details of the upgraded canopy physics parameterizations included in a specialized ammonia-only version of ACCESS (ACCESS-NH3). A Nomenclature list of all variables is provided at the end of the document.

2 Vertical Mixing and Transport

2.1 Eddy Diffusivity Parameterizations

Within the canopy, the turbulent eddy diffusivity profile, K_v^{cnpy}, is computed with a formulation derived from Raupach (1989)

$$K_v^{cnpy}(z,t) = S_c\,\sigma_w^2(z,t)\,T_L(z,t). \tag{1}$$

The Lagrangian time scale is calculated as

$$T_L = \frac{0.3\,h_c}{u_*}, \tag{2}$$

and the square of the vertical velocity fluctuation standard deviation is estimated with

$$\sigma_w^2 = (1.25\;u_*)^2\,. \tag{3}$$

To estimate an eddy diffusivity profile in the canopy, we assume the friction velocity is a local value that can be estimated as a function of the mean wind speed at each level (Weber, 1999) ($u_* = 0.13\,\overline{u}$), which results in

$$K_v^{cnpy}(z,t) = 0.07\,S_c\,\overline{u}(z,t)\,h_c. \tag{4}$$

Above the canopy, the surface layer parameterization of Stull (1988) and Seinfeld and Pandis (1998) is used

$$K_v^{abv}(z,t) = \frac{\kappa u_*(z-d)}{\phi((z-d)/L)} \tag{5}$$

with

$$\phi((z-d)/L) = \begin{cases} 1 + 4.7((z-d)/L) & \dfrac{z-d}{L} > 0 \quad \text{stable} \\ \\ 1 & \dfrac{z-d}{L} = 0 \quad \text{neutral} \\ \\ (1 - 15((z-d)/L))^{-0.5} & \dfrac{z-d}{L} < 0 \quad \text{unstable.} \end{cases} \tag{6}$$

where d is the zero-plane displacement height, taken here as $d = 0.67h_c$.

For the Coweeta site, the Monin-Obukhov length scale, L, is calculated from measured quantities as

$$L = -\frac{u_*^3 c_p \rho_0 \Theta_v}{0.4 g H_v} \tag{7}$$

with

$$\Theta_v = \Theta(1 + 0.61 q_0) \tag{8a}$$

$$H_v = H + 0.61 c_p \Theta E. \tag{8b}$$

At the top of the canopy, $z = h_c$, continuity of the above- and in-canopy eddy diffusivities is required so that

$$K_v^{cnpy}(h_c, t) = K_v^{abv}(h_c, t), \tag{9}$$

which uniquely determines the within-canopy atmospheric stability scaling factor, S_c, thereby allowing K_v^{cnpy} to vary with atmospheric stability while maintaining continuity with K_v^{abv}. For sensitivity simulations, a sensitivity factor is applied to the entire eddy diffusivity profile within the computational domain after first computing the profile as a function of the prevailing atmospheric stability with the formulation above.

3 Canopy Morphology and Physics

3.1 Leaf Area Density Profile

An important input to an ACCESS-NH3 simulation is the leaf area density (LAD) profile. LAD (m^2 m^{-3}) is the volumetric density of leaf area (two-sided) within the canopy as a function of vertical extent within the canopy. LAD (i.e., $a(z)$) is provided in the canopy morphology input file and is closely tied to the defined computational grid for a particular ACCESS simulation. Tools are provided in ACCESS-NH3 to either interpolate actual measurements of LAD (or leaf area index (LAI)) to the computational grid or, if no actual data is available for a particular simulated location, to approximate the LAD profile based on the predominate plant species in the canopy.

3.2 Radiative Fluxes

Measurements of above-canopy incoming total solar insolation and photosynthetic photon flux density (PPFD) are partitioned into direct and diffuse visible and near-infrared components using the algorithms of Weiss and Norman (1985), resulting in estimates of R_{beam}^{PPFD}, R_{diff}^{PPFD}, R_{beam}^{NIR}, and R_{diff}^{NIR}. Using these above-canopy fluxes, the algorithms of Bodin and Franklin (2012) are then used to calculate attenuated radiation fluxes of PPFD and NIR absorbed by sunlit and shaded leaves throughout the canopy

$$R^{sun} = \frac{k_d}{\sqrt{1-\gamma}} R_{diff} + \frac{k_d}{\sqrt{1-r}} R_{sc}^{\uparrow} + \frac{k_d}{\sqrt{1-t}} R_{sc}^{\downarrow} + k_b R_{0,b} \tag{10}$$

$$R^{shd} = \frac{k_d}{\sqrt{1-\gamma}} R_{diff} + \frac{k_d}{\sqrt{1-r}} R_{sc}^{\uparrow} + \frac{k_d}{\sqrt{1-t}} R_{sc}^{\downarrow} \tag{11}$$

with,

$$R_{diff} = R_{0,d}(1-\rho_t)e^{-k_d L_c} \tag{12}$$

$$R_{sc}^{\uparrow} = R_{0,b}r\frac{e^{-k_b L_c} - e^{k_d L_c - (k_b+k_d)L_{tot}}}{k_d + k_b} \tag{13}$$

$$R_{sc}^{\downarrow} = R_{0,b}t\frac{e^{-k_b L_c} - e^{-k_d L_c}}{k_d - k_b}. \tag{14}$$

The fraction of sunlit and shaded leaves at a canopy depth L_c (as a cumulative leaf area index from canopy top) is given by

$$f_{sun} = e^{-k_b L_c} \tag{15a}$$
$$f_{shd} = 1 - f_{sun}. \tag{15b}$$

At each level i in the canopy, longwave radiation fluxes are calculated after Norman (1979) as

$$R_{LW,i}^{\uparrow} = R_{LW,i-1}^{\uparrow}e^{-k_d\Delta L_i} + (1-e^{-k_d\Delta L_i})\varepsilon_{leaf}\sigma T_{l,i-1}^4 \tag{16a}$$
$$R_{LW,i}^{\downarrow} = R_{LW,i+1}^{\downarrow}e^{-k_d\Delta L_i} + (1-e^{-k_d\Delta L_i})\varepsilon_{leaf}\sigma T_{l,i+1}^4 \tag{16b}$$

with the upwelling longwave flux from the ground surface determined from

$$R_{LW,grnd}^{\uparrow} = \varepsilon_{grnd}\sigma T_s^4 \tag{17}$$

and the effective downwelling flux from the sky is calculated as

$$R_{LW,sky}^{\downarrow} = \varepsilon_{sky}^{'}\sigma T_{a,ncnpy+1}^4. \tag{18}$$

Total radiation absorbed by the sunlit and shaded canopy fractions at each level is then assembled from the components as

$$R_{abs,i}^{sun} = \alpha_{ppfd}R_{PPFD,i}^{sun} + \alpha_{nir}R_{NIR,i}^{sun} + R_{LW,i}^{\uparrow} + R_{LW,i}^{\downarrow} \tag{19a}$$
$$R_{abs,i}^{shd} = \alpha_{ppfd}R_{PPFD,i}^{shd} + \alpha_{nir}R_{NIR,i}^{shd} + R_{LW,i}^{\uparrow} + R_{LW,i}^{\downarrow}. \tag{19b}$$

3.3 Mean Wind Speed

The mean wind speed within the canopy, $\overline{u}(z)$, is computed from the empirical relation as presented in Meyers et al. (1998)

$$\bar{u}(z) = \bar{u}(h_c)exp(-\lambda_{\bar{u}}(1-z/h_c)^{\beta_{\bar{u}}}), \tag{20}$$

where, $\bar{u}(h_c)$ is the mean wind speed at the canopy top, and $\lambda_{\bar{u}}$ and $\beta_{\bar{u}}$ are parameters that depend on the canopy morphology and LAI,

$$\lambda_{\bar{u}} = min\left(4, \begin{matrix} LAI & forest \\ 0.65LAI & other \end{matrix}\right) \tag{21}$$

$$\beta_{\bar{u}} = 1 - 0.25\,(P-1) \tag{22}$$

with $P = 1$ for a symmetric LAD vertical distribution, $= 2$ for a slightly skewed upward distribution, and $= 3$ for a forest-type distribution concentrated in the upper canopy.

3.4 Leaf Energy Balance

At each level in the canopy, leaf temperature is calculated separately for the sunlit and shaded fractions from a leaf energy balance as

$$\underset{In}{R_{leaf}^{abs} - L_{leaf}} \;=\; \underset{Out}{H_{leaf} + \lambda E_{leaf}}. \tag{23}$$

Or, in terms of leaf variables,

$$R_{leaf}^{abs} - 2\varepsilon_{leaf}\sigma T_l^4 = 2c_p g_h\,(T_l - T_a) + \lambda g_v \left(\frac{e_{sat}(T_{leaf}) - e_a}{p_a}\right). \tag{24}$$

At each canopy level, Eq. (24) is solved for T_l iteratively via a Newton-Raphson method for both sunlit and shaded leaves, using the appropriate absorbed radiation from Eqs.(19). The calculation of leaf temperatures throughout the canopy is closely tied to calculation of stomatal resistances, net photosynthesis, air temperatures and water vapor profiles. Calculations are performed in an iterative manner for all of these canopy profiles until convergence is achieved for a particular time step.

3.5 Canopy Resistances

3.5.1 Leaf Boundary-Layer Resistance, $r_b^{NH_3}$

The leaf quasi-laminar boundary layer resistance for NH_3, $r_b^{NH_3}$, is estimated by (Hicks et al., 1987)

$$r_b^{NH_3} = \frac{2}{\kappa u_*} Sc^{2/3}, \tag{25}$$

where Sc is the Schmidt number, $= \nu_{air}/\mathcal{D}_{NH_3}$. To apply Eq.(25) to a multilayer canopy (rather than an idealized "big-leaf"), we define u_* as a "layer" friction velocity proportional to the in-canopy mean wind speed as $u_*(z) = 0.13\overline{u}(z)$ (after Weber, 1999), which results in

$$r_b^{NH_3}(z) = \frac{38.46}{\overline{u}(z)} Sc^{2/3}. \tag{26}$$

3.5.2 Leaf Stomatal Resistance, $r_s^{NH_3}$

Several parameterization options are available for the leaf stomatal resistance. The default option is a Ball-Berry type of parameterization as formulated by Medlyn et al. (2011), which uses the net photosynthetic assimilation rate, A_{net}

$$g_s(z) = g_0 + 1.6\left(1 + \frac{g_1}{\sqrt{e_{sat}\,(T_a(z)) - e_a(z)}}\right)\frac{A_{net}(z)}{C_{CO_2}}. \tag{27}$$

Using $g_s(z)$, stomatal resistance for a particular chemical species is then calculated from

$$r_s^{NH_3}(z) = \frac{p_a/R_{gas}T_l(z)}{g_s(z)} \frac{\mathcal{D}_{H_2O}}{\mathcal{D}_{NH_3}}. \tag{28}$$

Other options for stomatal resistance are based on a Jarvis-type expression of the form (Jarvis, 1976)

$$r_s^{NH_3}(z) = \frac{r_{s,min}}{f(PPFD) \cdot f(T) \cdot f(VPD) \cdot f(\psi_l)} \frac{\mathcal{D}_{H_2O}}{\mathcal{D}_{NH_3}}. \tag{29}$$

Eq.(29) can be applied to any canopy type with appropriate choices of input parameters as recommended by Zhang et al. (2003).

The PPFD correction function is defined as

$$f(PPFD) = \left(1 + \frac{b_{rs}}{PPFD}\right)^{-1}, \tag{30}$$

where PPFD and b_{rs} have units μ mol m^{-2} s^{-1}, and b_{rs} is specific to a particular vegetative canopy (Zhang et al., 2003) .

The temperature correction function is defined as

$$f(T) = \frac{T - T_{min}}{T_{opt} - T_{min}} \left[\frac{T_{max} - T}{T_{max} - T_{opt}}\right]^{\frac{T_{max}-T_{opt}}{T_{opt}-T_{min}}}, \tag{31}$$

with values of T_{min}, T_{max}, and T_{opt} as recommended by Zhang et al. (2003).

The water vapor pressure deficit correction function is given by

$$f(VPD) = 1 - b_{vpd}VPD \tag{32}$$

where,

$$VPD = e_{sat}(T_a) - e_a. \tag{33}$$

Finally, the water stress correction function is defined by

$$f(\psi_l) = \frac{\psi_l - \psi_{c_2}}{\psi_{c_1} - \psi_{c_2}}, \tag{34}$$

with

$$\psi_l = -0.72 - 0.0013R_{\downarrow}. \tag{35}$$

ACCESS-NH3 uses the Medlyn et al. (2011) formulation as the default parameterization for stomatal resistance; however, the Jarvis-type of formulation can be employed via compile-time code modifications, if desired.

3.5.3 Leaf Mesophyllic and Cuticular Resistances

In ACCESS-NH3, two NH_3-specific choices are provided for the cuticular resistance. The default comes from Flechard et al. (2010) and is given by

$$r_w = min\left(r_{w_{max}}, r_{w_{min}}\ e^{\alpha_f(100-RH)}\ e^{\beta_f|T_a^C|}\right). \tag{36}$$

where, α_f = 0.11, β_f = 0.15, $r_{w_{min}}$ = 0.10 s cm^{-1}, and $r_{w_{max}}$ = 12.0 s cm^{-1}.

The second option, from Massad et al. (2010), accounts for the ambient molar ratio of total acid to NH_3

$$r_w = \frac{0.315}{AR}\ e^{\alpha_m(100-RH)}\ e^{\beta_m|T_a^C|}\ LAI^{-0.5} \tag{37}$$

where, α_m = 0.148, β_m = 0.15, and

$$AR = \frac{2[SO_2] + [HNO_3] + [HCl]}{[NH_3]}, \tag{38}$$

where, $[SO_2]$, $[HNO_3]$, $[HCl]$, and $[NH_3]$ are the molar ambient air concentrations of sulfur dioxide, nitric acid, hydrochloric acid and ammonia, respectively.

The choice of which option for r_w to use for a particular simulation is currently made at compile time.

3.6 Photosynthetic Assimilation

The net photosynthetic assimilation rate, A_{net}, is needed in the Medlyn et al. (2011) calculation of stomatal conductance (Eq.(27)). The formulation of Campbell and Norman (1998) as derived from Collatz et al. (1991) is used

$$A_{net} = A - R_d \tag{39}$$

$$A = \frac{J_p + J_s - \sqrt{(J_p + J_s)^2 - 4\beta_t J_p J_s}}{2\beta_t} \tag{40}$$

$$J_p = \frac{J_E + J_C - \sqrt{(J_E + J_C)^2 - 4\theta_t J_E J_C}}{2\theta_t} \tag{41}$$

$$J_C = \frac{V_m(C^i_{co_2} - \Gamma^*)}{C^i_{co_2} + K_c(1 + C^a_{o_2}/K_0)} \tag{42}$$

$$J_E = \frac{\alpha_{PPFD} e_m PPFD(C^i_{co_2} - \Gamma^*)}{C^i_{co_2} + 2\Gamma^*} \tag{43}$$

$$J_s = V_m/2 \tag{44}$$

$$V_m = \frac{V_{m,25}\ exp\left[0.088(T_l^C - 25)\right]}{1 + exp\left[0.29(T_l^C - 41)\right]} \tag{45}$$

$$R_d = \frac{R_{d,25}\ exp\left[0.069(T_l^C - 25)\right]}{1 + exp\left[1.3(T_l^C - 55)\right]} \tag{46}$$

$$\Gamma^* = \frac{C^a_{o_2}}{2\tau} \tag{47}$$

$$\tau = \tau_{25}\ exp(-0.056(T_l^C - 25)) \quad (48)$$

$$K_c = K_{c,25}\ exp(0.074(T_l^C - 25)) \quad (49)$$

$$K_0 = K_{0,25}\ exp(0.018(T_l^C - 25)). \quad (50)$$

Parameter	Value	Units	Description
$V_{m,25}$	54.0	μmol/m^2-s	maximum Rubisco capacity @ 25°C
$R_{d,25}$	1.5	μmol/m^2-s	leaf respiration rate @ 25°C
$K_{c,25}$	300	μmol/mol	Michaelis constant for CO_2 @ 25°C
$K_{0,25}$	300000	μmol/mol	inhibition constant for O_2 @ 25°C
$C_{o_2}^a$	210000	μmol/mol	O_2 mixing ratio
τ_{25}	2600	mmol/mmol	CO_2/O_2 specificity ratio
β_t , θ_t	0.98, 0.95	-	colimitation transition parameters
α_{PPFD}	0.8	-	PPFD absorptivity of a leaf
e_m	0.08	mol/mol	molecules CO_2 fixed/quantum of absorbed radiation

Table 1: Parameters used in the photosynthesis model.

$C_{co_2}^i$ in these equations is the intercellular CO_2 concentration (μmol/mol), which is calculated using the Medlyn et al. (2011) expression

$$C_{co_2}^i = C_{co_2}^a \left(\frac{g_1}{g_1 + \sqrt{VPD}} \right). \quad (51)$$

Since $PPFD$ and T_l^C both vary vertically within the canopy, $A_{net} = A_{net}(z,t)$. The parameters used in Eqs.(39-51) are given in Table(1) and are derived from Campbell and Norman (1998), Collatz et al. (1991), and Houborg et al. (2009) (for a temperate deciduous forest).

3.7 Air Temperature and Humidity

Previous versions of ACCESS have used simple assumptions (e.g., linear interpolation) to approximate the vertical distributions of air temperature and humidity within the canopy based on measurements made in or above the canopy. In ACCESS-NH3, an option is provided to deterministically simulate within-canopy air temperature and humidity profiles via coupled energy and mass balance equations adapted from Bonan et al. (2018).

Air temperature within the canopy is determined from

$$\rho c_p \frac{\partial T_a(z,t)}{\partial t} + \frac{\partial H_a(z,t)}{\partial z} = S_H(z,t) \quad (52)$$

where, the turbulent vertical flux of sensible heat, H, is given by

$$H_a(z,t) = \overline{w' T_a'}(z,t) \quad (53)$$

and again using gradient diffusion theory as was done with tracer transport

$$H_a(z,t) = -\rho c_p K_v(z,t) \frac{\partial T_a(z,t)}{\partial z}. \quad (54)$$

The term $S_H(z,t)$ in Eq.(52) is the volumetric source/sink of sensible heat from/to the canopy (J m^{-3} s^{-1}) and is apportioned between the sunlit and shaded portions of the canopy (where $a(z)$ is the leaf area density (m^2 m^{-3}) profile of the canopy)

$$S_H(z,t) = \left[H_l^{sun}(z,t)f_{sun}(z,t) + H_l^{shd}(z,t)(1 - f_{shd}(z,t))\right] a(z) \tag{55}$$

which gives

$$\rho c_p \frac{\partial T_a(z,t)}{\partial t} = \frac{\partial}{\partial z}\left(\rho c_p K_v(z,t)\frac{\partial T_a(z,t)}{\partial z}\right) + \left[H_l^{sun}(z,t)f_{sun}(z,t) + H_l^{shd}(z,t)(1 - f_{shd}(z,t))\right] a(z) \tag{56}$$

with the sunlit and shaded sensible heat fluxes (J m^{-2} s^{-1}) given by

$$H_l^{sun}(z,t) = 2c_p(T_l^{sun}(z,t) - T_a(z,t))g_b(z,t) \tag{57a}$$

$$H_l^{shd}(z,t) = 2c_p(T_l^{shd}(z,t) - T_a(z,t))g_b(z,t). \tag{57b}$$

Similarly, water vapor concentration within the canopy is governed by

$$\frac{\partial q(z,t)}{\partial t} + \frac{\partial E_q(z,t)}{\partial z} = S_E(z,t) \tag{58}$$

with the turbulent vertical flux of water vapor expressed as

$$E_q(z,t) = \overline{w'q'}(z,t) = -\rho K_v(z,t)\frac{\partial(q(z,t)/\rho)}{\partial z} \tag{59}$$

and the volumetric source/sink of water vapor from/to the canopy (mol H_2O m^{-3} s^{-1}) as

$$S_E(z,t) = \left[E_l^{sun}(z,t)f_{sun}(z,t) + E_l^{shd}(z,t)(1 - f_{shd}(z,t))\right] a(z). \tag{60}$$

The governing equation for water vapor in the canopy is then

$$\frac{\partial q(z,t)}{\partial t} = \frac{\partial}{\partial z}\left(\rho K_v(z,t)\frac{\partial(q(z,t)/\rho)}{\partial z}\right) + \left[E_l^{sun}(z,t)f_{sun}(z,t) + E_l^{shd}(z,t)(1 - f_{shd}(z,t))\right] a(z) \tag{61}$$

with the sunlit and shaded water vapor fluxes (mol H_2O m^{-2} s^{-1}) given by

$$E_l^{sun}(z,t) = \left[q_{sat}(T_l^{sun}(z,t)) - q(z,t)\right] g_l^{sun}(z,t) \tag{62a}$$

$$E_l^{shd}(z,t) = \left[q_{sat}(T_l^{shd}(z,t)) - q(z,t)\right] g_l^{shd}(z,t). \tag{62b}$$

The air temperature and water vapor profiles are intimately connected via boundary conditions. At the upper boundary of the computational domain, z_N, the air temperature and water vapor boundary conditions are set using measurements made above the canopy. If observed fluxes of sensible and latent heat are available, then the upper boundary conditions are given by

$$K_v(z_N,t)\frac{\partial T_a(z_N,t)}{\partial z} = -\frac{H^{obs}(z_N,t)}{\rho C_p} \quad (63)$$

$$K_v(z_N,t)\frac{\partial q(z_N,t)}{\partial z} = -E^{obs}(z_N,t) \quad (64)$$

where, H^{obs} and E^{obs} $(= LE^{obs}/\lambda)$ are measured sensible and water vapor fluxes at the top of the canopy. If sensible heat and latent heat fluxes are not available, then the upper boundary conditions are set as

$$T_a(z_N,t) = T_a^{ref}(t) \quad (65)$$

$$q(z_N,t) = q^{ref}(t) \quad (66)$$

where, T_a^{obs} and q^{obs} are air temperature and water vapor concentration (i.e., humidity) measured above the canopy or extrapolated from measured values to the top of the computational domain using standard atmosphere lapse rates.

At the surface, the boundary condition for air temperature is derived from the surface energy balance

$$R_n = H + \lambda E + G \quad (67)$$

or

$$R_n = c_p(T_s - T_a^o)g_{bg} + \lambda(\alpha_{soil}q_{sat}(T_s) - q(0,t))v_s + \frac{K^{soil}}{\Delta z_{soil}}(T_s - T_s^{\Delta z}). \quad (68)$$

After some rearrangement, Eq.(68) can be written as

$$T_a^o = T_a(0,t) = T_s - (R_n - \lambda(\alpha_{soil}q_{sat}(T_s) - q(0,t))v_s - \frac{K^{soil}}{\Delta z_{soil}}(T_s - T_s^{\Delta z}))/c_p g_{bg} \quad (69)$$

where it is seen that the air temperature surface boundary condition, $T_a(0,t)$, is dependent on the water vapor concentration at the surface, $q(0,t)$. Because of this dependence, the water vapor profile must first be obtained before the air temperature integration can be performed.

For water vapor, the surface boundary condition is given by

$$-\rho K_v(0,t)\frac{\partial(q(0,t)/\rho)}{\partial z} = -v_s(q(0,t) - \alpha_{soil}q_{sat}(T_s)). \quad (70)$$

The final equations are then: for T_a, Eqs.(56-57) with boundary conditions Eq.(65) and Eq.(69); for q, Eqs.(61-62) with boundary conditions Eq.(66) and Eq.(70).

In the ACCESS-NH3 simulation control file, a user can choose to either solve these deterministic equations for T_a and/or q or use linear interpolation from available measurements to approximate the vertical profiles of either of these variables.

4 Surface Exchange

The soil-atmosphere exchange velocity for ammonia, $v_s^{NH_3}$, is needed to calculate the exchange of NH_3 between the surface and atmosphere. In the case of Coweeta, measurements indicate that little NH_3 exists in the soil, but substantial amounts occur in the litter on the forest floor. In this case, we use a formulation for $v_s^{NH_3}$ as

$$v_s^{NH_3}(t) = \frac{1}{r_{bg} + r_{litter,NH_3}}. \tag{71}$$

The soil boundary layer resistance, r_{bg} is based on the formulation and data of Schuepp (1977),

$$r_{bg} = \frac{Sc - ln(\delta_0/z_l)}{\kappa u_*^g}. \tag{72}$$

Although Schuepp (1977) provides an expression for the calculation of δ_0, it is clear from trying to reproduce the results in the article that a constant value of $\delta_0/z_l = 0.018$ was used to produce the results of Scheupp's Table II. Schuepp (1977) also provides an expression for u_*^g based on his laboratory experiments. Here we assume that an approximation of $u_*^g = 0.13\overline{u}$ after the recommendation of Weber (1999) is sufficient. With these simplifications and recognizing that $Sc \approx 0.7$, Scheupp's expression becomes

$$r_{bg} = 88.7/\overline{u}. \tag{73}$$

Few studies have attempted to measure litter resistance, and as far as the authors are aware none have measured the resistance of NH_3 transport through litter on a deciduous forest floor. As a result, r_{litter,NH_3} is assumed in ACCESS-NH3 for the Coweeta study and adjusted as necessary to reproduce the measured NH_3 gradients near the surface.

Nomenclature

Acronyms

BC	boundary conditions
BVOC	biogenic volatile organic compounds
LAD	leaf area density (m^2 m^{-3} or cm^2 cm^{-3})
LAI	leaf area index (m^2 m^{-2} or cm^2 cm^{-2})
LW	long-wave radiation
NIR	near-infrared radiation
ODE	ordinary differential equations
PFT	plant functional type
PPFD	photosynthetic photon flux density (μmol m^{-2} s^{-1})
RH	relative humidity ()
VPD	vapor pressure deficit (kPa)

Greek Symbols

α_{NIR}	leaf absorptivity of NIR radiation
α_{PPFD}	leaf absorptivity of PPFD radiation
α_f	relative humidity coefficient in Flechard et al. (2010) (%$^{-1}$)
α_{soil}	fractional relative humidity in the soil pore space adjacent to the free water level
β_f	temperature coefficient in Flechard et al. (2010) (C^{-1})

β_t, θ_t	colimitation transition parameters for calculation of photosynthetic assimilation rate
γ	leaf scattering coefficient $= t + r$ (0.27 for PPFD; 0.69 for NIR)
Γ^*	CO_2 compensation point (μmol mol^{-1})
δ_0	from Schuepp (1977), the distance above the ground surface where molecular and turbulent transport are similar in magnitude (m)
ΔL_i	incremental change in cumulative leaf area density at level i in the canopy (m^{-2} m^3)
Δz_{soil}	soil depth over which the soil temperature gradient is calculated (m)
$\varepsilon_{sky}^{'}$	effective emissivity of the sky as suggested by Crawford and Duchon (1999) and Prata (1996)
ε_{grnd}	emissivity of the ground surface
ε_{leaf}	emissivity of leaves
Θ_v	potential virtual temperature (K)
Θ	potential temperature $= T(p_0/p)^{R/c_p}$ (K)
κ	von Karman's constant (= 0.4)
λ	molar heat of evaporation for water vapor (J mol^{-1})
$\lambda_{\bar{u}}$, $\beta_{\bar{u}}$	parameters used in the Meyers et al. (1998) canopy wind speed parameterization
λE	latent heat flux (W m^{-2})
λE_{leaf}	latent heat exchange between the leaf and the surrounding air (W m^{-2})
ν_{air}	kinematic viscosity of air (cm^2 s^{-1})
ρ	molar density of air (mol m^{-3})
ρ_0	molar density of air at the surface (mol m^{-3})
ρ_t	overall canopy reflectance, $= (\frac{1-\sqrt{1-\gamma}}{1+\sqrt{1-\gamma}})(\frac{2}{1+1.6cos\Phi)})$
σ_w	standard deviation of the vertical velocity fluctuation (cm s^{-1})
σ	Stefan-Boltzmann constant $= 5.67 \times 10^{-8}$ W m^{-2} K^{-4}
τ	CO_2/O_2 specificity ratio in the photosynthetic assimilation rate parameterization (mmol/mmol)
τ_{25}	CO_2/O_2 specificity ration at 25°C (mmol/mmol)
ψ_{c_1}, ψ_{c_2}	canopy-specific empirical constants (MPa)
ψ_l	leaf water potential (MPa)
ψ_m, ψ_h	stability functions from Bonan (2016)

Roman Symbols

$\overline{w'q'}$	turbulent vertical flux of water vapor (mol m^2 s^{-1})
$\overline{w'T_a'}$	turbulent vertical flux of sensible heat (W m^{-2})
A	gross photosynthesis assimilation rate (μmol m^{-2} s^{-1}) defined by the minimum between J_s and J_p with colimitation transition parameter β_t
A_{net}	net photosynthesis assimilation rate (μ mol m^{-2} s^{-1})

b_{vpd}	canopy-specific empirical constant (kPa^{-1})
$C^a_{o_2}$	O_2 mixing ratio (μmol mol^{-1}) = 210,000
$C^a_{co_2}$	atmospheric CO_2 mixing ratio (μ mol mol^{-1})
$C^i_{co_2}$	intercellular CO_2 mixing ratio (μmol mol^{-1}) $= C^a_{co_2}\left(\frac{g_1}{g_1+\sqrt{e_s(T_l)-e_a}}\right)$ from Medlyn et al. (2011)
c_p	specific heat capacity of air (J mol^{-1} K^{-1})
$\mathcal{D}_{H_2O}$	molecular diffusivity of water vapor in air (cm^2 s^{-1})
$\mathcal{D}_{NH_3}$	molecular diffusivity of NH_3 in air (cm^2 s^{-1})
E	water vaporization flux (mol H_2O m^{-2} s^{-1})
E^{obs}	water vapor flux measured at the top of the canopy (mol H_2O m^{-2} s^{-1})
E_l^{sun}, E_l^{shd}	water vapor fluxes between the air and sunlit and shaded portions of the canopy (mol H_2O m^{-2} s^{-1})
e_a	water vapor pressure of surrounding air (kPa)
e_m	maximum quantum efficiency as molecules CO_2 fixed per quantum of absorbed radiation = 0.08 mol mol^{-1}
E_q	vertical turbulent flux of water vapor (mol H_2O m^{-2} s^{-1})
E_{leaf}	water vapor flux between the leaf and the canopy air (mol H_2O m^{-2} s^{-1}
e_{sat}	saturation water vapor pressure at given temperature (kPa)
$f(\psi_l)$	water stress correction function for a Jarvis-type stomatal resistance
$f(PPFD)$	PPFD correction function for a Jarvis-type stomatal resistance
$f(T)$	temperature correction function for a Jarvis-type stomatal resistance
$f(VPD)$	water vapor pressure deficit (VPD) correction function for a Jarvis-type stomatal resistance
f_{shd}	fraction of canopy that is shaded
f_{sun}	fraction of canopy that is sunlit
G	ground heat flux (W m^{-2})
g	gravitational acceleration (9.822 m s^{-2})
g_l^{sun}, g_l^{shd}	leaf water vapor conductance for sunlit and shaded canopy fractions (m s^{-1})
g_0, g_1	empirical parameters specific to particular vegetative canopies for the Medlyn et al. (2011) stomatal conductance parameterizations
g_b	leaf boundary layer conductance (mol m^{-2} s^{-1})
g_h	conductance for sensible heat between a leaf and the surrounding air (mol m^{-2} s^{-1})
g_s	stomatal conductance for water vapor (mol m^{-2} s^{-1})
g_v	overall leaf conductance for water vapor (mol m^{-2} s^{-1})
g_{bg}	soil boundary layer conductance (mol m^{-1} s^{-1})
H	sensible heat flux (W m^{-2})
H^{obs}	sensible heat flux measured at the top of the canopy (W m^{-2})

H_l^{sun}, H_l^{shd}	sensible heat fluxes between sunlit and shaded portions of the canopy and the surrounding air (J m^{-2} s^{-1})
H_a	vertical turbulent flux of sensible heat (J m^{-2} s^{-1})
h_c	canopy height (cm)
H_v	virtual sensible heat flux (J m^{-2} s^{-1})
H_{leaf}	sensible heat exchange between a leaf and the surrounding air (W m^{-2})
J_C	Rubisco-limited photosynthetic assimilation rate (μmol m^{-2} s^{-1})
J_E	light-limited photosynthetic assimilation rate (μmol m^{-2} s^{-1})
J_p	minimum between J_E and J_C with transition abruptness parameter θ_t (μmol m^{-2} s^{-1})
J_s	sucrose-limited photosynthetic assimilation rate (μmol m^{-2} s^{-1})
K_v^{abv}	above-canopy eddy diffusivity profile (cm^2 s^{-1})
K_v^{cnpy}	within-canopy eddy diffusivity profile (cm^2 s^{-1})
K^{soil}	thermal conductivity of soil at measured water content (W m^{-1} K^{-1})
K_0	inhibition constant for O_2 in the photosynthetic assimilation rate parameterization (μmol mol^{-1})
k_b	extinction coefficient for direct beam radiation $= \frac{\sqrt{x^2+tan^2\Phi}}{x+1.774(x+1.182)^{-0.733}}$, for an ellipsoidal leaf angle distribution (Campbell and Norman, 1998)
K_c	Michaelis constant for CO_2 in the photosynthetic assimilation rate parameterization (μmol mol^{-1})
k_d	extinction coefficient for diffuse radiation (0.68 for PPFD; 0.45 for NIR)
K_v	turbulent eddy diffusivity (cm^2 s^{-1})
$K_{0,25}$	inibition constant for O_2 at 25°C (μmol mol^{-1})
$K_{c,25}$	Michaelis constant for CO_2 at 25°C (μmol mol^{-1})
L	Monin-Obukhov length scale (m)
L_c	cumulative leaf area index (m^2 m^{-2})
L_{leaf}	longwave radiation emitted by the leaf (W m^{-2})
L_{tot}	total leaf area index (m^2 m^{-2})
LE	latent heat flux = λE (J m^{-2} s^{-1})
p_a	air pressure (kPa)
q	molar water vapor concentration (mol m^{-3})
$q^{'}$	turbulent fluctuation of molar water vapor concentration (mol m^{-3})
q^{obs}	molar water vapor concentration at $z = z_N$ (mol m^{-3})
q_0	specific humidity at the surface (kg kg^{-1})
q_{sat}	saturation molar water vapor concentration at the given temperature (T_l^{sun} or T_l^{shd}) (mol m^{-3})
r	leaf reflectance (0.11 for PPFD; 0.43 for NIR)
$R_{LW}^{\downarrow}$	downwelling longwave radiation (W m^{-2})
$R_{sc}^{\downarrow}$	downwelling scattered PPFD (μmol m^{-2} s^{-1}) or NIR (W m^{-2})

$R^{\uparrow}_{LW,grnd}$	upwelling longwave radiation from the ground (W m^{-2}
$R^{\uparrow}_{LW}$	upwelling longwave radiation (W m^{-2})
$R^{\uparrow}_{sc}$	upwelling scattered PPFD (μmol m^{-2} s^{-1}) or NIR (W m^{-2})
R^{abs}_{leaf}	radiation absorbed by a leaf $= PPFD + NIR + LW$ (W m^{-2})
$r_b^{NH_3}$	leaf boundary layer resistance for NH_3 (s cm^{-1})
$r_s^{NH_3}$	leaf stomatal resistance for NH_3 (s cm^{-1})
R^{NIR}_{beam}	estimated direct beam NIR (W m^{-2})
R^{NIR}_{diff}	estimated diffuse NIR (W m^{-2})
R^{PPFD}_{beam}	estimated direct beam PPFD (μmol m^{-2} s^{-1})
R^{PPFD}_{diff}	estimated diffuse PPFD (μmol m^{-2} s^{-1})
R^{shd}_{abs}	total absorbed radiation on shaded leaves (W m^{-2})
$R^{sun,shd}_{NIR}$	NIR radiation in sunlit or shaded fractions (W m^{-2})
$R^{sun,shd}_{PPFD}$	PPFD radiation in sunlit or shaded fractions (W m^{-2})
R^{sun}_{abs}	total absorbed radiation on sunlit leaves (W m^{-2})
R_d	leaf respiration rate (μmol m^{-2} s^{-1})
R_n	net radiation at the surface (SW+LW) (W m^{-2})
r_w	NH_3-specific cuticular resistance (s cm^{-1})
$R_{0,b}$	above canopy direct beam PPFD (μmol m^{-2} s^{-1}) or NIR (W m^{-2})
$R_{0,d}$	above canopy diffuse PPFD (μmol m^{-2} s^{-1}) or NIR (W m^{-2})
$R_{\downarrow}$	total solar irradiance (W m^{-2})
r_{bg}	resistance to diffusion across the quasi-laminar boundary layer next to the soil surface (s cm^{-1})
$R_{d,25}$	$= R_d$ at 25° C = 1.5 μmol m^{-2} s^{-1}
R_{diff}	incoming diffuse PPFD (μmol m^{-2} s^{-1}) or NIR (W m^{-2})
R_{gas}	ideal gas constant (atm m^{-3} mol^{-1} K^{-1})
r_{litter,NH_3}	resistance to diffusion through the litter layer on the forest floor (s cm^{-1})
$r_{s,min}$	minimum leaf stomatal resistance (s cm^{-1})
S_c	atmospheric stability scaling factor
$S_E(z,t)$	volumetric source/sink of water vapor from/to the canopy (mol H_2O m^{-3} s^{-1})
$S_H(z,t)$	volumetric source/sink of sensible heat from/to the canopy (J m^{-3} s^{-1})
t	leaf transmittance (0.16 for PPFD; 0.26 for NIR)
T_a^0	effective air temperature at the ground surface (K)
$T_a^{'}$	turbulent fluctuation of air temperature (K)
$T_s^{\Delta z}$	soil temperature at a depth Δz_{soil} below the surface (K)

T_a^C	air temperature (C)
T_l^C	leaf temperature (C)
T_a^{obs}	air temperature at $z = z_N$ (K)
T_l^{sun}, T_l^{shd}	leaf temperature in the sunlit and shaded portions of the canopy (K)
T_a	air temperature (K)
T_L	Lagrangian time scale (s)
T_s	ground surface temperature (K)
$T_{a,ncnpy+1}$	above canopy air temperature (K)
T_l	leaf temperature (K)
$\bar{u}$	mean wind speed (cm s^{-1})
u_g^*	parameterized friction velocity defined by Schuepp (1977)
u_*	friction velocity (cm s^{-1})
u_{ref}	mean wind speed at $z = z_{ref}$ (m s^{-1})
V_m	maximum Rubisco capacity in the photosynthetic assimilation rate parameterization (μmol m^{-2} s^{-1})
v_s	soil-atmosphere exchange velocity of water vapor (cm s^{-1})
$V_{m,25}$	$= V_m$ at 25° C = 100 μmol m^{-2} s^{-1}
$w^{'}$	turbulent fluctuation of vertical wind (cm s^{-1})
x	leaf angle distribution parameter (canopy specific)
z_l	distance from the ground surface where the mean wind speed profile can be represented by a lognormal profile (m)
z_N	height of the top of the model domain (cm)
a(z)	leaf area density vertical profile within the canopy (m^2 m^{-3})
d	zero-plane displacement height (cm)
P	empirical constant in the Meyers et al. (1998) canopy wind speed parameterization that accounts for canopy morphology
Sc	Schmidt number $= \nu_a/\mathcal{D}$

References

Bodin, P. and O. Franklin (2012), "Efficient modeling of sun/shade canopy radiation dynamics explicitly accounting for scattering." *Geoscientific Model Development*, 5, 535–541.

Bonan, G. (2016), *Ecological Climatology*. Cambridge University Press, New York, NY.

Bonan, G., E. Patton, I. Harman, K. Oleson, J. Finnigan, Y. Lu, and E. Burakowski (2018), "Modeling canopy-induced turbulence in the Earth system: a unified parameterization of turbulent exchange within plant canopies and the roughness sublayer (CLM-ml v0)." *Geoscientific Model Development*, 11, 1467–1496.

Campbell, G. S. and J. M. Norman (1998), *An Introduction to Environmental Biophysics*. Springer, New York, NY.

Collatz, G. J., J. T. Ball, C. Grivet, and J. A. Berry (1991), "Physiological and environmental regulation of stomatal conductance, photosynthesis and transpiration: a model that includes a laminar boundary layer." *Agricultural and Forest Meteorology*, 54, 107–136.

Crawford, T. M. and C. E. Duchon (1999), "An improved parameterization for estimating effective atmospheric emissivity for use in calculating daytime downwelling long wave radiation." *Journal of Applied Meteorology*, 48, 474–480.

Flechard, C. R., C. Spirig, A. Neftel, and C. Ammann (2010), "The annual ammonia budget of fertilised cut grassland - Part 2: Seasonal variations and compensation point modeling." *Biogeosciences*, 7, 537–556.

Hicks, B. B., D. D. Baldocchi, T. P. Meyers, R. P. Hosker, Jr., and D. R. Matt (1987), "A preliminary multiple resistance routine for deriving dry deposition velocities from measured quantities." *Water, Air, and Soil Pollution*, 36, 311–330.

Houborg, R., M. C. Anderson, J. M. Norman, T. Wilson, and T. Meyers (2009), "Intercomparison of a 'bottom-up' and 'top-down' modeling paradigm for estimating carbon and energy fluxes over a variety of vegetative regimes across the U. S." *Agricultural and Forest Meteorology*, 149, 1875–1895.

Jarvis, P. G. (1976), "The interpretation of the variations in leaf water potential and stomatal conductance found in canopies in the field." *Philosophical Transactions of the Royal Society of London B*, 273, 593–610.

Massad, R.-S., E. Nemitz, and M. A. Sutton (2010), "Review and parameterisation of bi-directional ammonia exchange between vegetation and the atmosphere." *Atmospheric Chemistry and Physics*, 10, 10359–10386.

Medlyn, B. E., R. A. Duursma, D. Eamus, D. S. Ellsworth, I. C. Prentice, C. V. M. Bartons, K. Y. Crous, P. de Angelis, M. Freeman, and L. Wingate (2011), "Reconciling the optimal and empirical approaches to modeling stomatal conductance." *Global Change Biology*, 17, 2134–2144.

Meyers, T. P., P. Finkelstein, J. Clarke, T. G. Ellestad, and P. F. Sims (1998), "A multilayer model for inferring dry deposition using standard meteorological measurements." *Journal of Geophysical Research*, 103, 22645–22661.

Norman, J. M. (1979), "Modeling the Complete Crop Canopy." In *Modification of the Aerial Environment of Crops* (B. J. Barfield and J. F. Gerber, eds.), 1 edition, 249–277, American Society of Agricultural Engineers, St. Joseph, MI.

Prata, A. J. (1996), "A new long-wave formula for estimating downward clear-sky radiation at the surface." *Quarterly Journal of the Royal Meteorological Society*, 122, 1127–1151.

Raupach, M. R. (1989), "Applying Lagrangian fluid mechanics to infer scalar source distributions from concentration profiles in plant canopies." *Agricultural and Forest Meteorology*, 47, 85–108.

Saylor, R. D. (2013), "The Atmospheric Chemistry and Canopy Exchange Simulation System (ACCESS): model description and application to a temperate deciduous forest canopy." *Atmospheric Chemistry and Physics*, 13, 693–715.

Schuepp, P. H. (1977), "Turbulent transfer at the ground: On verification of a simple predictive model." *Boundary-Layer Meteorology*, 12, 171–186.

Seinfeld, J. H. and S. N. Pandis (1998), *Atmospheric Chemistry and Physics: From Air Pollution to Climate Change*. John Wiley & Sons, New York, NY.

Stull, R. B. (1988), *An Introduction to Boundary Layer Meteorology*. Kluwer Academic Publishers, Dordrecht, The Netherlands.

Weber, R. O. (1999), "Remarks on the definition and estimation of friction velocity." *Boundary-Layer Meteorology*, 93, 197–209.

Weiss, A. and J. M. Norman (1985), "Partitioning solar radiation into direct and diffuse, visible and near-infrared components." *Agricultural and Forest Meteorology*, 34, 205–213.

Zhang, L., J. R. Brook, and R. Vet (2003), "A revised parameterization for gaseous dry deposition in air quality models." *Atmospheric Chemistry and Physics*, 3, 2067–2082.
